# Colorectal cancer cell line-derived organoid model with stem cell properties captures the regrowing state of residual cancer cells after neoadjuvant chemotherapy

**DOI:** 10.1038/s41420-025-02567-w

**Published:** 2025-06-20

**Authors:** Kiyotaka Nakano, Eiji Oki, Masaki Yamazaki, Masami Suzuki, Shigeto Kawai, Takanori Fujita, Atsuhiko Kato, Yoko Zaitsu, Tomoko Jogo, Chie Kato, Takeshi Watanabe, Eri Hashimoto, Chiyoko Nishime, Etsuko Fujii, Koji Ando, Genta Nagae, Norifumi Harimoto, Mitsuhiko Ota, Hiroshi Saeki, Hiroyuki Aburatani, Yoshihiko Maehara, Tatsumi Yamazaki

**Affiliations:** 1 Translational Research Division, Chugai Pharmaceutical Co., Ltd., Chugai Life Science Park Yokohama 216 Totsuka-cho, Totsuka-ku, Yokohama, Kanagawa 244-8602 Japan; 2 Department of Surgery and Science, Graduate School of Medical Sciences, Kyushu University, 3-1-1 Maidashi, Higashi-ku, Fukuoka-shi, Fukuoka, 812-8582 Japan; 3 Central Institute for Experimental Medicine and Life Science, 3-25-12 Tonomachi, Kawasaki-ku, Kawasaki, 210-0821 Japan; 4 Genome Science Division, Research Center for Advanced Science and Technology, The University of Tokyo, 4-6-1 Komaba, Meguro-ku, Tokyo, 153-8904 Japan; 5 Chugai Research Institute for Medical Science, Inc., Chugai Life Science Park Yokohama 216 Totsuka-cho, Totsuka-ku, Yokohama, Kanagawa 244-8602 Japan; 6 Kyushu Central Hospital of the Mutual Aid Association of Public School Teachers, 3-23-1 Shiobaru, Minami-ku, Fukuoka-shi, Fukuoka, 815-8588 Japan; 7 Chugai Pharmaceutical Co., Ltd., 1-1 Nihonbashi-Muromachi 2-chome, Chuo-ku, Tokyo, 103-8324 Japan

**Keywords:** Translational research, Colorectal cancer, Cancer stem cells

## Abstract

The effectiveness of colorectal cancer (CRC) therapy is limited owing to the absence of treatments targeting drug-tolerant residual cancer cells. Although neoadjuvant therapy is effective, pathological examination of residual tumors has revealed the presence of small clusters of LGR5-positive cancer cells in the fibrous tissue. Here, we established a colorectal cancer cell line-derived organoid (CCD-organoid) regrowth model using a patient-derived cell line with cancer stem cell properties and demonstrated that it displayed the morphological characteristics of small clusters in clinical tissues. Time course analysis of single-cell RNA sequencing of the CCD-organoid regrowth model revealed various states and dynamic alterations within non-cycling cells. We identified subpopulations highly expressing protein translation-related genes *RPL17* and *EEF1G*. To identify key signals for the transition of residual cancer cells to regrowth, we evaluated inhibitors targeting pathways such as the Wnt pathway, reactive oxygen species pathway, and RNA polymerase I pathway, highlighted in the single-cell RNA sequencing analysis. Only the polymerase I-inhibitor BMH-21 significantly reduced tumor growth both in vitro and in vivo, indicating the critical cell subpopulation driving recurrence. Our results demonstrate the possibility of a unique therapeutic target for CRC treatment targeting drug-tolerant residual cancer cells.

## Introduction

Colorectal cancer (CRC) is the third most commonly diagnosed malignancy and the second leading cause of cancer-related deaths worldwide [1]. The current standard of care is surgery; however, chemotherapy, radiotherapy, and targeted therapies are often employed in cases where surgery is unfeasible or in combination with surgery. These strategies have contributed to improved survival, particularly in patients with early-stage disease [2, 3**]**. However, the effectiveness of standard adjuvant therapy remains limited, and recurrence and metastasis caused by residual cancer cells remain major issues in cancer therapy.

The persistence of chemo-resistant tumor cells is referred to as minimal residual disease (MRD). Monitoring MRD through circulating tumor DNA analysis is useful for evaluating the risk of recurrence in CRC [4]. However, the precise characteristics of residual cancer cells remain unclear, and effective drugs targeting their elimination remain to be identified. The mechanisms underlying drug resistance include clonal selection and drug-induced mechanisms [5]. The clonal selection mechanism is driven by genetic diversity, in which certain clones with advantageous mutations survive and proliferate in response to drug pressure. As a drug-induced non-genetic mechanism, the presence of a subpopulation that evades drug damage by transitioning to a non-cycling state has garnered interest [6, 7**]**. The relationship between drug-tolerant residual cancer cells and cancer stem cell (CSC) properties has attracted attention, with the presence of CSC markers such as LGR5- and CD44-positive cells being reported in colorectal cancer [8–11]. Recently, adaptation of ovarian cancer cells to cancer therapy and by extension, resistance has been reported [12]. Nevertheless, comprehensive analyses detailing the state of residual cancer cells in clinical tumors remain relatively scarce.

Colorectal cancer cell line-derived organoid (CCD-organoid) cultures of patient-derived cancer cells can be effectively used to distinguish differences in sensitivity to anticancer drugs [13, 14]. Additionally, they are useful for understanding mechanisms underlying treatment resistance and metastasis, and single-cell RNA-seq allows for the analysis of the heterogeneous state of cancer at the single-cell level [15**–**17]. Understanding the transition from a dormant state to regrowth is crucial for therapeutic targeting. However, a reproducible in vitro system capable of detailed analysis of this process has not yet been reported.

We previously established an in vitro colorectal cancer cell line, PLR123, from a patient-derived xenograft [18]. PLR123 is considered to possess CSC properties owing to the expression of CSC markers, its high tumor-initiating activity in mouse xenograft studies, and the hierarchical structure of the engrafted tumors resembles clinical cancers. Additionally, PLR123 can form homogeneous ductal structures in three-dimensional culture, suggesting that PLR123 is useful as a highly reproducible CCD-organoid model [19, 20]. The PLR123 cancer cell line was originally established from PDX and forms similar morphological characteristics to those of clinical tumors under 3D culture conditions. As PLR123 has been maintained as a cell line, we have coined the term “colorectal cancer cell line-derived organoids (CCD-organoids)” for 3D structures in this paper.

In this study, we focused on the recurrence of residual cancer cells following chemotherapy in CRC. Initially, we performed a detailed pathological analysis of the state of residual cancer cells using tissues from clinical colorectal cancer neoadjuvant cases. Subsequently, we constructed a reproducible regrowth model using PLR123 CCD-organoids to mimic the regrowth of residual cancer cells through non-genetic resistance mechanisms. By performing time-series single-cell RNA sequencing and evaluating chemical inhibitors using the reproducible regrowth model, we successfully identified critical phases in the recurrence of residual cancer cells.

## Results

### Existence of small clusters in fibrous tissues following drug treatment in neoadjuvant chemotherapy patients

**Fig. 1:**
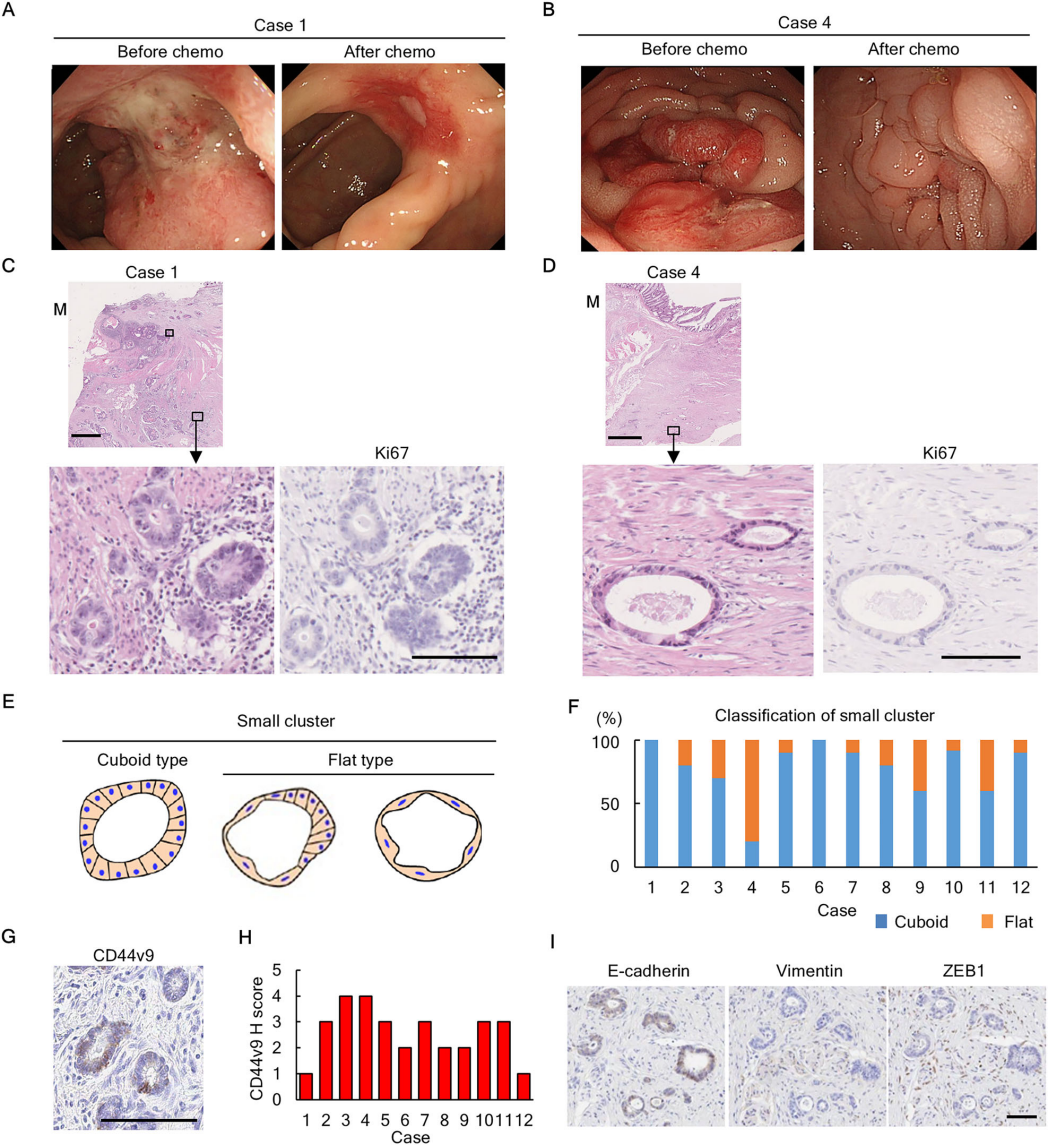
Presence of small clusters of cancer cells exist in resected tumor tissues following chemotherapy. **A**, **B** Colonoscopy before and after chemotherapy revealing a marked decrease in primary tumor size (A, Case 1; B, Case 4). **C**, **D** Representative images of histological findings after chemotherapy in clinical CRC (HE, Ki-67) (C, Case 1; D, Case 4). Small clusters contain no Ki-67-positive cells. Scale bars: 1 mm (low magnification) and 100 μm (high magnification). **E** The classification schema for small clusters, cuboid type, and flat type (two examples). **F** Percentage of classification of small clusters (cuboid and flat type) in 12 cases. Blue, cuboid; Orange, flat. **G** Representative image of CD44v9 positive cells. Scale bar: 100 μm. **H** H-score of CD44v9 in 12 cases. **I** Representative images for the expression of EMT-relating proteins (E-cadherin, Vimentin, and ZEB1). Scale bar: 100 μm.

We performed a pathological analysis of tissues collected during surgery after chemotherapy in clinical CRC cases where neoadjuvant therapy was effective (Fig. 1A, B). The number of cancer nests with glandular structures characteristic of adenocarcinoma were reduced. These regions were extensively replaced by fibrous tissue (Fig. 1C, D). In the fibrous tissue regions of all cases, a few residual cancer cells remained and formed small clusters mainly composed of a single layer of epithelial cells (Fig. 1C, D**and** Fig. S1A). These small clusters exhibited wide morphological variation, with compositions ranging from tall columnar cells (cuboid type, Fig. 1C) to flat cells (flat type, Fig. 1D); the cuboid type predominated (Fig. 1E, F).

For molecular pathology analysis, Ki-67-positive cells were detected in glandular structures **(**Fig. S1B); however, most small cluster structures were Ki-67 negative, indicating that they were non-proliferative (Fig. 1C, D**)**. The cancer stem marker CD44v9 was expressed at various levels in small cluster constituent cells in all cases, indicating that these clusters were composed of cells with stemness properties (Fig. 1G, H). The small clusters maintained epithelial characteristics with E-cadherin expression and did not express typical epithelial-mesenchymal transition markers such as Vimentin and ZEB1 (Fig. 1I).

**Fig. 2:**
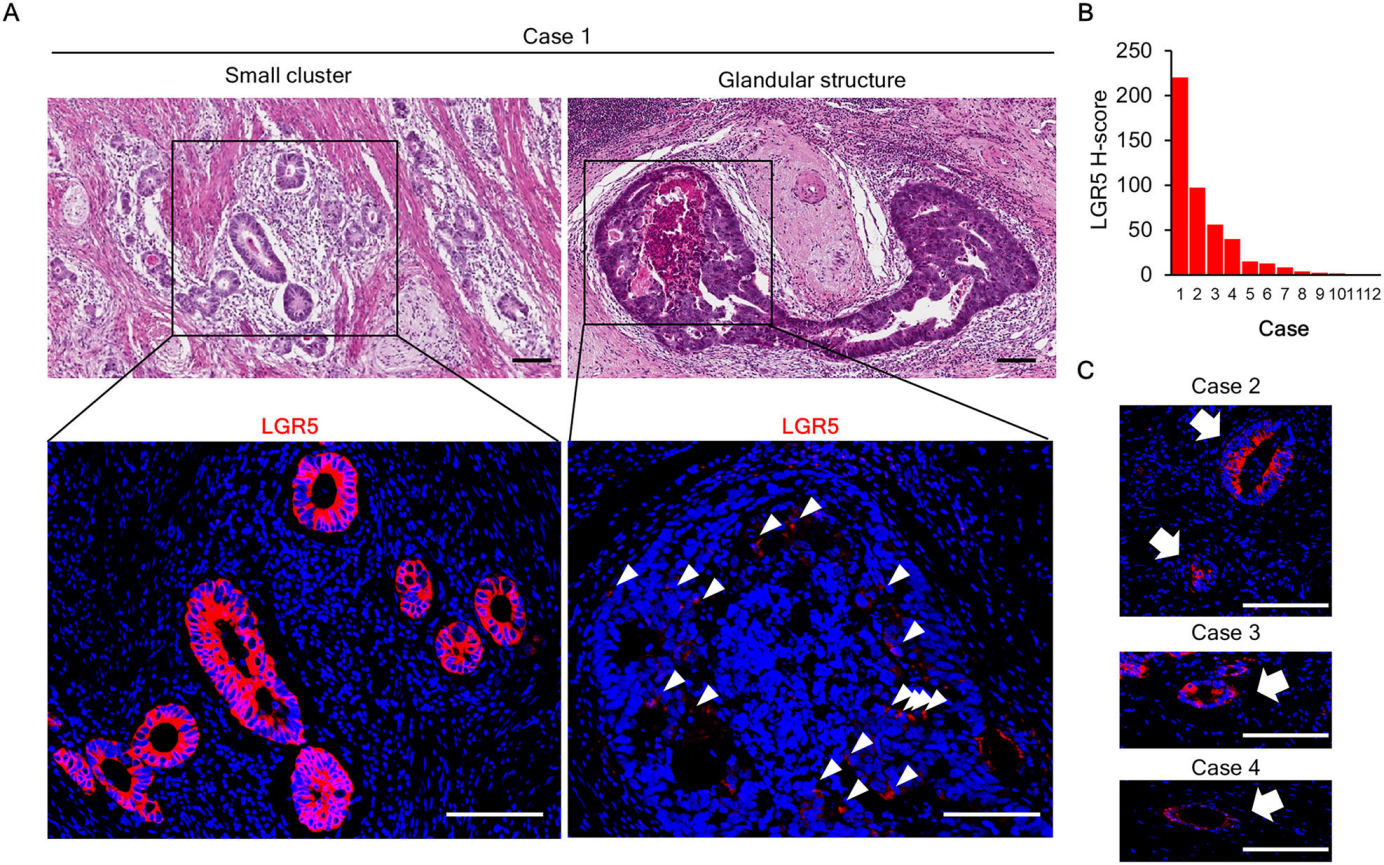
Presence of small clusters of cells with LGR5 expression in resected tumor tissue following chemotherapy. **A** Representative image for LGR5 expression in a small cluster in Case 1. Arrowheads indicate LGR5-expression in glandular structure. Scale bar: 100 μm. **B** H-score of LGR5 for 12 cases. **C** Representative image for LGR5 expression in a small cluster (arrow) in Cases 2–4. Scale bar: 100 μm.

Next, because stemness properties were observed in the small cluster constituent cells, we analyzed the expression of the CRC stem cell marker LGR5 using a specific antibody against LGR5 through intensive immunofluorescence staining for low-expression proteins. High expression was observed in 4 of 12 cases and positive expression in 7 of the remaining 8 cases (Fig. 2A–C, Fig. S1C). In Case 1, the expression characteristics differed based on the structure; high LGR5 expression frequency and intensity were observed in the small cluster structures, whereas scattered positive cells were observed in the glandular structures (Fig. 2A). These results indicated that cells in various states existed in the tumor tissues following chemotherapy, and cancer stem-like cells were identified among the residual tumor cells.

### Establishment of an in vitro CCD-organoid-based regrowth model

**Fig. 3:**
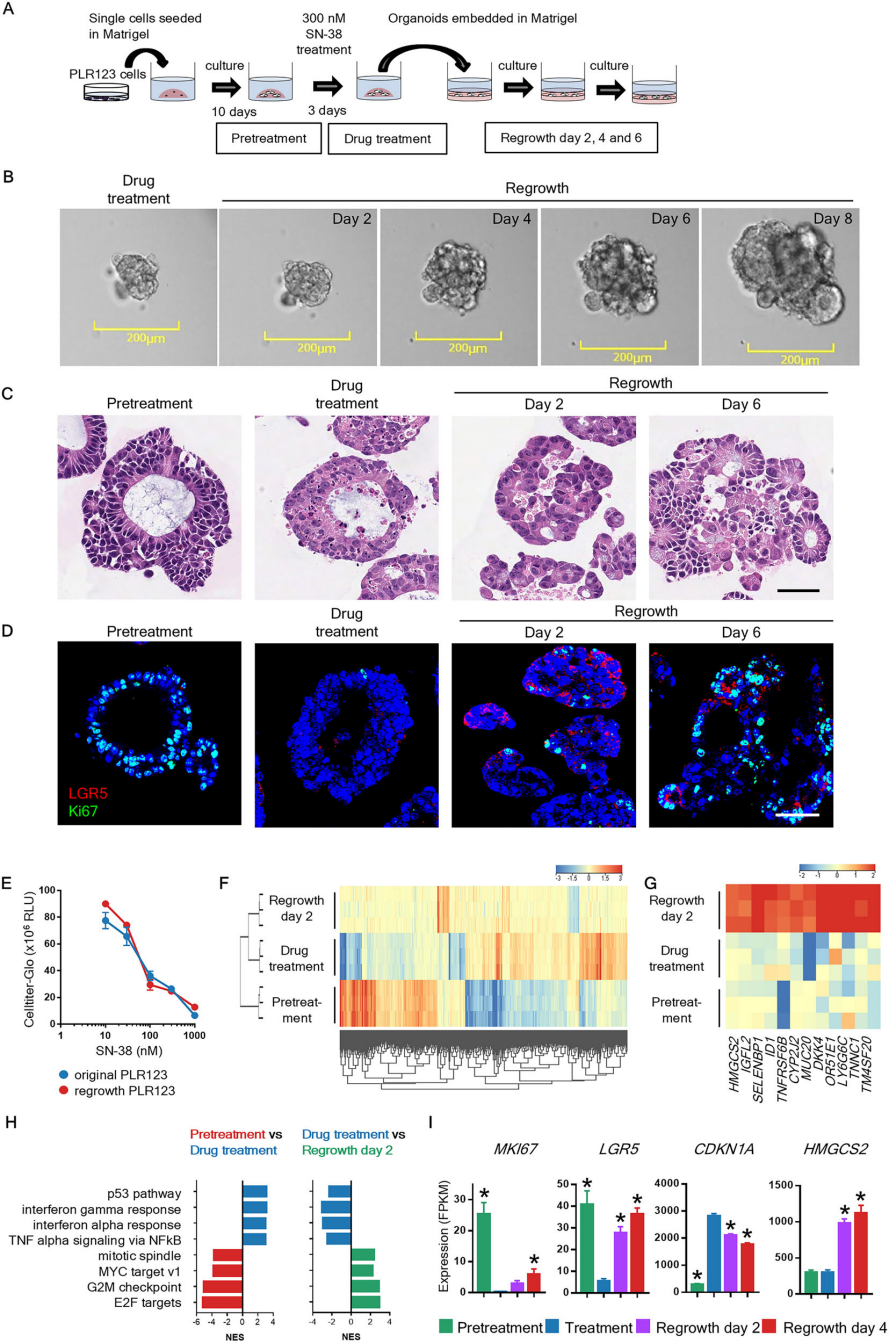
Establishment of a CCD-organoid model recapitulating the regrowth of DTP cells. **A** Experimental design for CCD-organoid regrowth after SN-38 treatment. **B** Bright-field images of whole PLR123 CCD-organoids following SN-38 treatment. Scale bars: 200 μm. **C** Histopathological images of regrown CCD-organoids. Scale bar: 50 μm. **D** Expression of LGR5 and Ki-67 in the regrowth phase. Red, LGR5; green, Ki67; blue, DAPI. Scale bars: 50 μm. **E** Comparison of the sensitivity to SN-38 of the original and regrown PLR123 cells. **F** Hierarchical clustering of transcriptional profiles of pretreated and drug-treated cells and cells on regrowth day 2 (*n* = 3). **G** Gene Set Enrichment Analysis (GSEA) using hallmark gene sets from the Molecular Signatures Database (MSigDB). Strongly enriched signatures were selected and placed in order of normalized enrichment scores. **H** Heatmap of the expression of genes selectively expressed on regrowth day 2. **I** Changes in *MKI67*, *LGR5*, *CDKN1A*, and *HMGCS2* expression. *P < 0.05 vs. treatment group. Data are shown as the mean ± standard deviation. Independent samples *t* tests were performed for comparisons of the means between two groups, and Dunnett’s test was used to compare the means of multiple groups with that of the control group.

To model the process of residual tumor cells transitioning to recurrence after chemotherapy, we established an in vitro regrowth model using a CCD-organoid derived from LGR5-positive PLR123 CRC cells. The PLR123 cells displayed morphological features of CRC in three-dimensional culture in Matrigel, including epithelial cell polarity and luminal structures (Fig. S2A–E). After 3 days of treatment with 300 nM of the irinotecan metabolite SN-38, numerous cells survived. When the remaining cell aggregates were collected, re-embedded in Matrigel, and cultured in drug-free conditions, they resumed proliferation (Fig. 3A, B). In the drug treatment group, a large number of cells in the lumen and between remaining cells exhibited nuclear pyknosis. Additionally, immunohistochemical staining confirmed that the remaining cells were Ki67 negative. However, small cell aggregates were observed on regrowth day 2, some of which contained LGR5- and Ki-67-positive cells (Fig. 3C, D, Fig. S2F, G). By day 6, numerous cells had become Ki67-positive, and small luminal structures had formed. The percentages of LGR5-positive cells were 4.1%, 14.7%, 58.5%, and 37.5% for pretreatment, drug treatment, day 2, and day 6, respectively (Fig. 3D). This regrowth reflected the characteristics of the clinical tissues. The sensitivity of regrown PLR123 cells to SN-38 was equivalent to that of the original PLR123 cells, suggesting that the resistance mechanism is not genetic but involves reversible changes in proliferation and quiescence (Fig. 3E).

Bulk RNA sequencing (RNA-seq) at various time points revealed substantial differences in expression and characteristic gene expression profiles at each stage (Fig. 3F, G). SN-38 treatment suppressed the expression of genes encoding proliferative signals, such as *MKI67*, and enhanced that of genes encoding p53 signals, including *CDKN1A*. The expression of these genes reverted to pretreatment levels during the regrowth phase. Genes specifically upregulated during the regrowth phase included *HMGCS2*, which is involved in ketone synthesis, and genes encoding mucin and metabolic enzymes related to intestinal function (Fig. 3H, I). Similar changes in gene expression were also observed in the two-dimensional cultured parent PLR123 cells **(**Fig. S2H**)**.

**Fig. 4:**
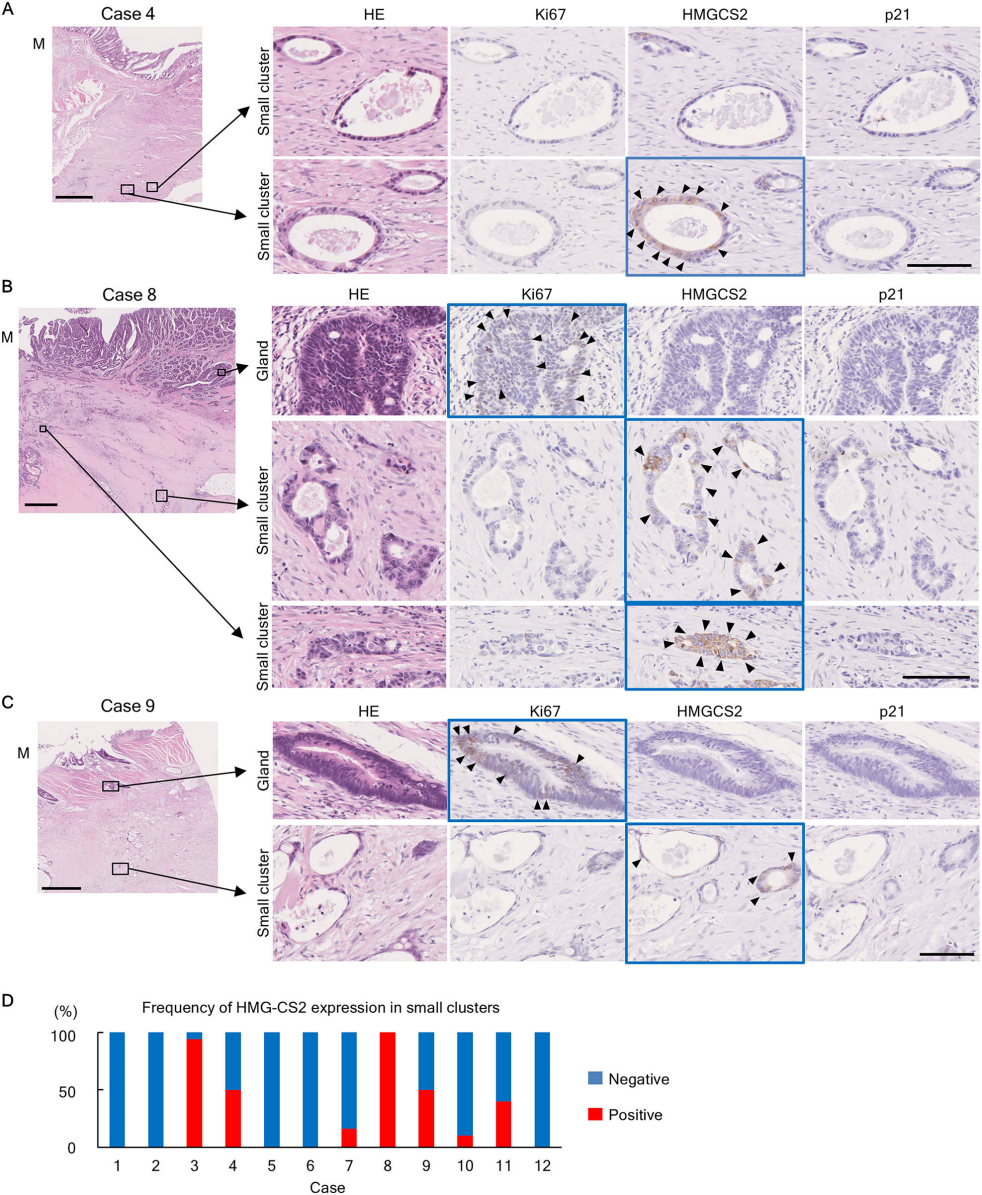
Expression of HMGCS2 in clinical CRC cases. **A** Case 4, (**B**) Case 8, and (**C**) Case 9. Arrowheads indicate Ki-67- and HMGCS2-positive cells. The blue outlines indicate regions where positive cells are present. The expression of HMGCS2 tends to be relatively abundant in the small cluster cuboid type and is complementary to the Ki-67 positive regions. Scale bar: 100 μm. **D** Frequency of HMGCS2-expression in small clusters. Red, percentage of HMGCS2-positive small clusters; Blue percentage of HMGCS2-negative small clusters.

To confirm protein expression in addition to gene expression, the CCD-organoids were immunohistochemically stained for Ki-67 and HMGCS2. HMGCS2 was the most strongly upregulated in bulk RNA-seq, revealing that HMGCS2-positive cells differed from cells strongly positive for the proliferation marker Ki-67 and the DNA damage marker p21 (Fig. S3). Next, we analyzed the regrowth CCD-organoid model and its clinical relevance using molecular pathology (Fig. 4). In residual small cluster cells of the clinical CRC neoadjuvant therapy cases analyzed earlier, p21-negative states were observed in all cases, presumably because the patient tissues were collected at least 3 weeks after the final anticancer drug administration. Small cluster cells were negative for Ki67, but HMGCS2 expression with various staining profiles (diffuse to sporadic) was present in 7 of 12 cases although not all small clusters expressed HMGCS2 (Fig. 4D). In addition, HMGCS2 was expressed in small clusters, as well as glandular structures (Fig. S4). As in the in vitro regrowth model, non-cycling residual cells in clinical cases after chemotherapy also had multiple cell states, leading to further deep analysis of this CCD-organoid regrowth model to reveal the characteristics of residual cells.

### Time-series analysis of single-cell (sc) RNA-seq data from CCD-organoids

To analyze the process of CCD-organoid regrowth at the single-cell level, scRNA-seq was performed. Data from 15,144 cells were obtained for each of five points: pretreatment, during drug treatment, and on regrowth days 2, 4, and 6 (Fig. S5A). The data for each time point were merged, batch-corrected, and visualized using Uniform Manifold Approximation and Projection (UMAP).

**Fig. 5:**
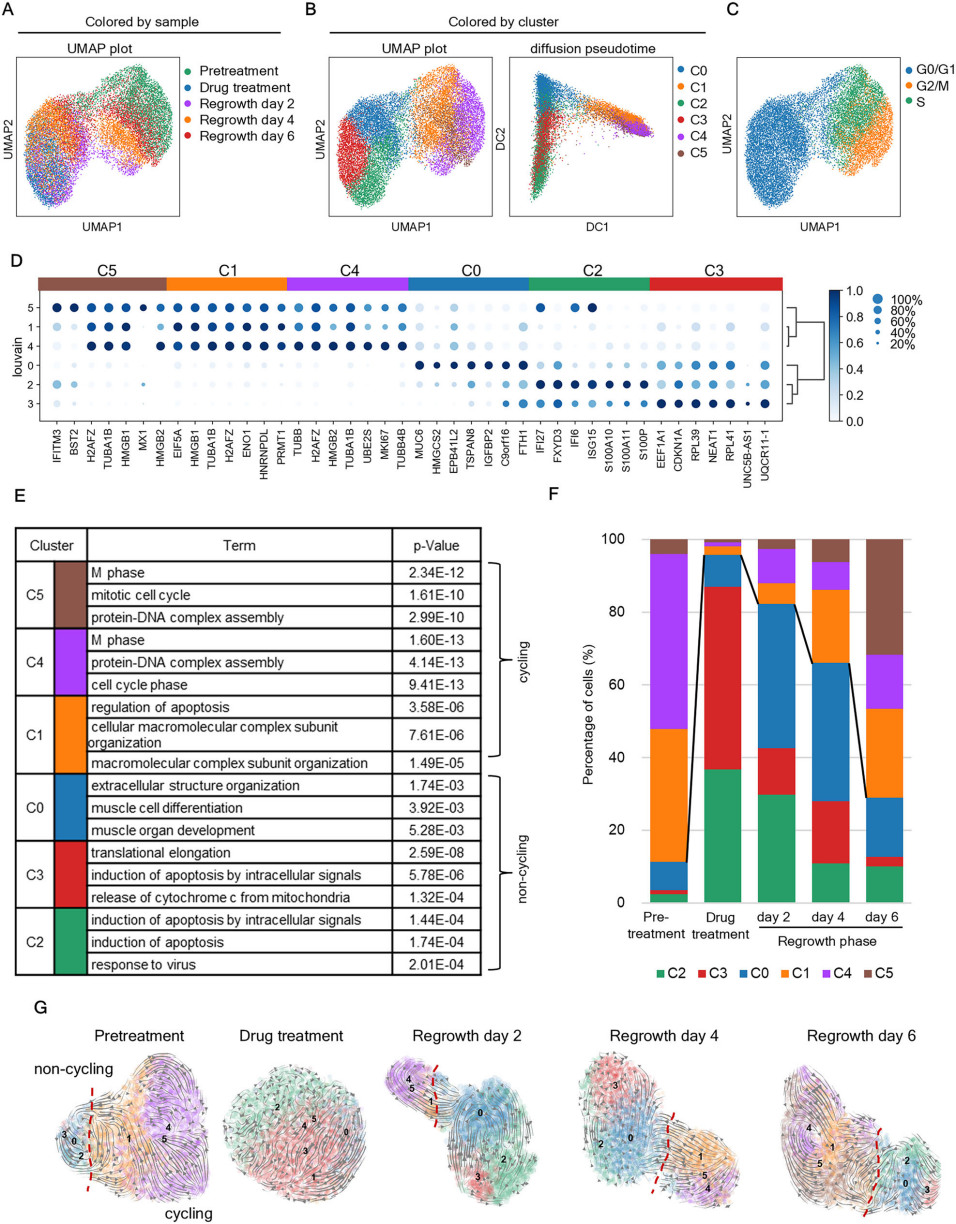
Analysis of the cancer cell status shifting toward regrowth using scRNA-seq. **A–C** UMAP visualization of 15,114 cells from PLR123 CCD-organoids. Cells are colored by sample (**A**), cluster (**B**), or cell cycle (**C**). A diffusion pseudotime plot colored by cluster is shown in (**B**). **D** Dot plot of cluster-specific genes. The color represents the mean expression within each cluster, and the dot size indicates the fraction of positive cells in the cluster. **E** Gene ontology analysis of differentially expressed genes in each cluster. **F** Relative abundances of the cell types observed at each time point. **G** Velocity field projected onto the UMAP plot of scRNA-seq data from pretreated and drug-treated cells and cells on regrowth days 2, 4, and 6. The red dotted line delineates cycling from non-cycling cells.

Based on the expression profiles, six clusters were identified (Fig. 5A–C, Fig. S5B). Dot plots of characteristic genes and pathway analysis results for each cluster are shown in Fig. 5D, E, S5C. The differentially expressed genes for each cluster are listed in Table S2. The six clusters were classified into two categories: cycling (C1, C4, and C5) and non-cycling (C0, C2, and C3). Among the non-cycling clusters, C0 exhibited high expression of *HMGCS2* and *MUC6*. In C2 and C3, apoptotic signaling was activated, and C3 showed high expression of *CDKN1A* and genes encoding ribosomal proteins and translation factors.

When we examined the composition ratio at each time point, the proportions of C2 and C3 were highest in drug-treated samples, suggesting that these cells were strongly affected by DNA damage (Fig. 5F). The proportion of C0 was the highest on regrowth day 2 and decreased as the proportion of cycling clusters increased. Individual data at each time point were projected using UMAP (Fig. 5G). The red dotted line in the figure delineates non-cycling from cycling cells. The pretreatment samples were visualized separately in UMAP (Fig. S6). This confirmed that they were composed of various cells, including MKI67-positive and CDKN1A/HMGCS2-positive cells. The results of RNA velocity analysis indicate dynamic changes between each cluster.

### Trajectory analysis of non-cycling cancer cells on regrowth day 2

**Fig. 6:**
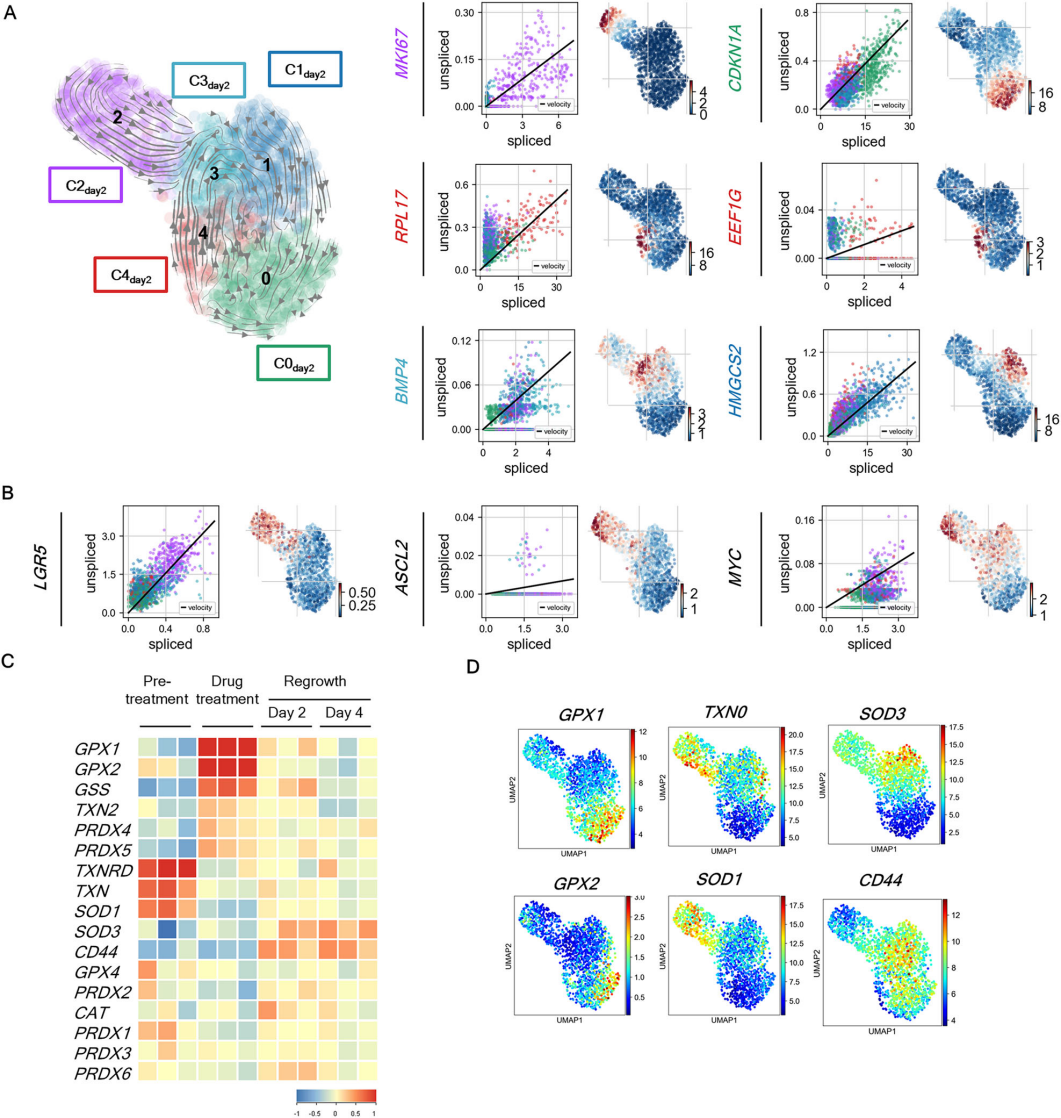
Analysis of regrowth day 2 scRNA-seq data reveals pathway differences among clusters. **A** UMAP visualization of 1698 cells on regrowth day 2. **A**, **B** Summary of RNA velocity analysis of the indicated genes. The left plots show the ratio variation between unspliced (Y-axis) and spliced (X-axis) mRNA levels for the indicated genes. **C** Heatmap derived from bulk RNA-seq profiles of PLR123 CCD-organoids at various time points (pretreatment, after drug treatment, on regrowth days 2 and 4) for genes related to ROS control. **D** UMAP plot of the expression of genes related to ROS control.

We focused on regrowth day 2 to capture the early changes in regrowth and analyzed the cell signals activated in each cluster in detail. Data from regrowth day 2 cells were classified into five clusters, which we labeled C0_day2_ to C4_day2_ (Fig. 6A, Fig. S7A). C2_day2_ cells were in a proliferative state; C0_day2_ cells highly expressed the DNA damage marker CDKN1A; and C4_day2_ cells highly expressed genes involved in protein translation, such as *RPL17* and *EEF1G*. *HMGCS2* was characteristic gene in C1_day2_. Non-cycling cells near C2_day2_ in the UMAP plot expressed Wnt signaling-related genes, such as *LGR5*, *ASCL2*, and *MYC* (Fig. 6B). C4_day2_ cells were in a state of active protein synthesis, as confirmed by pathway analysis (Fig. S7B), and were located upstream of C3_day2_ cells in the RNA velocity plot. C1_day2_ cells, which were HMGCS2-positive, were thought to transition from C2_day2_ and C3_day2_ to CDKN1A-positive C0_day2_ based on the RNA velocity analysis results.

Bulk RNA-seq and scRNA-seq analyses confirmed the altered expressions of ROS-related genes, which are reportedly associated with drug resistance (Fig. 6C, D). The glutathione-related genes *GPX1* and *GPX2* were highly expressed in drug-treated cells and tended to be highly expressed in C0_day2_ cells. The thioredoxin-related genes *TXN* and *SOD1* were highly expressed in the pretreatment stage and the proliferative C3_day2_ cluster. By contrast, *SOD3* and *CD44*, which are involved in glutathione uptake, were highly expressed in the regrowth phase and C3_day2_ and C1_day2_ cells.

The expression patterns of ROS-related genes varied greatly depending on the timing and cluster; this analysis revealed the overall group of genes that fluctuated in expression during the early stages of regrowth.

### In vitro evaluation of survival and regrowth of CCD-organoids by chemical inhibitors

**Fig. 7:**
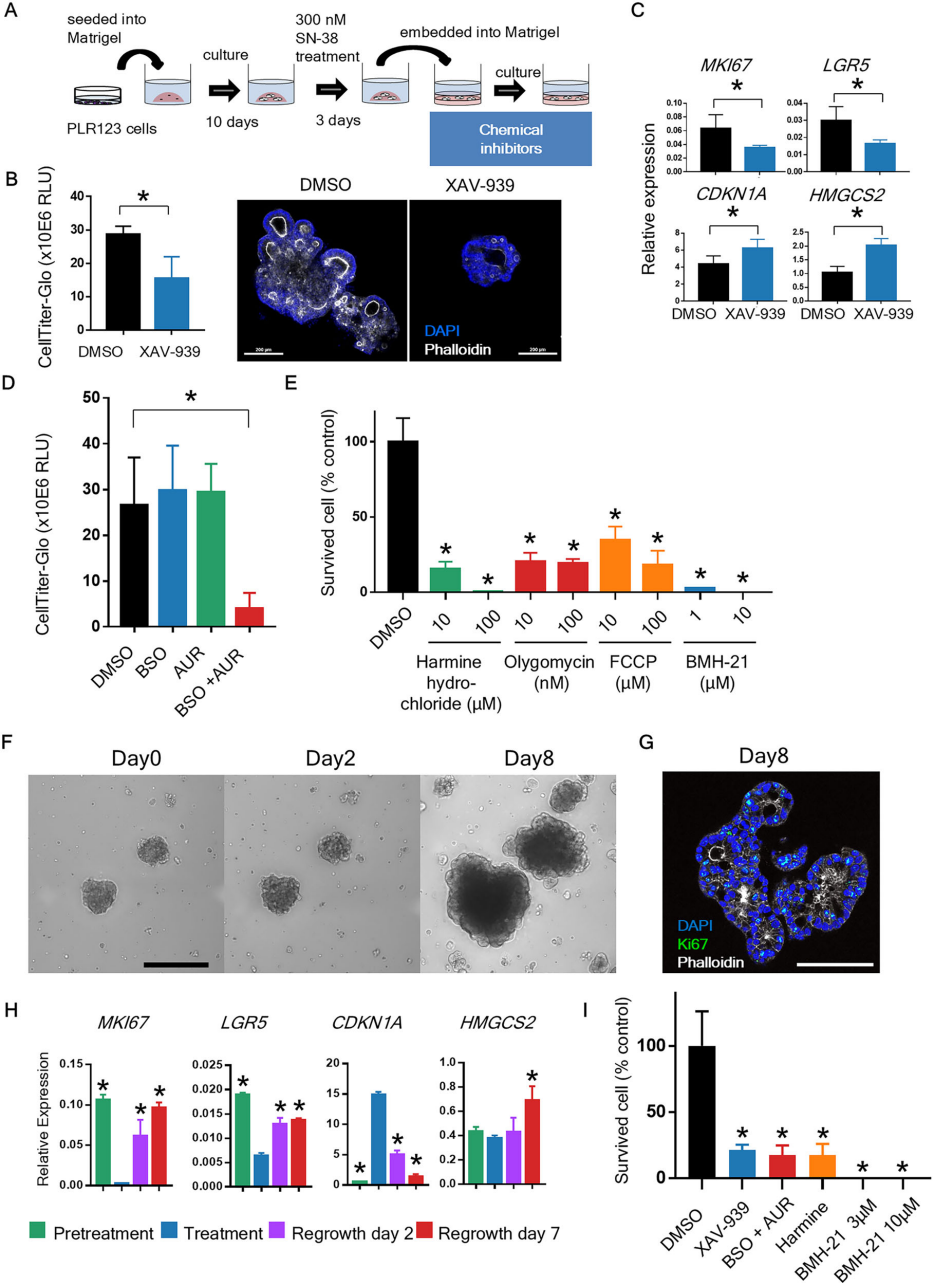
Identification of critical pathways through treatment of PLR123 CCD-organoid regrowth models with chemical inhibitors. **A** Experimental design. **B** Cell viability on regrowth day 12 after XAV-939 (10 μM) treatment (*n* = 6). The right panel shows a whole-mount image of an CCD-organoid on regrowth day 12. White, phalloidin; blue, DAPI. Scale bars: 200 μm. **C** Changes in RNA expression after XAV-939 treatment. RNA samples were collected on regrowth day 6 (*n* = 3). **D** Cell viability in PLR123 CCD-organoids on regrowth day 6 after L-buthionine sulfoximine (200 μM) and auranofin (200 nM) treatment (*n* = 4). **E** Cell viability in PLR123 CCD-organoids on regrowth day 10 after harmine hydrochloride, oligomycin, phenylhydrazone, or BMH-21 treatment. **F** Bright-field images of LS174T CCD-organoids after 300 nM SN-38 treatment. Scale bars: 200 μm. **G** Whole-mount immunostaining image of CCD-organoids on regrowth day 8. Green, Ki67; white, phalloidin; blue, DAPI. Scale bars: 200 μm. **H** Changes in *MKI67*, *LGR5*, *CDKN1A*, and *HMGCS2* expression. **I** Cell viability in LS174T CCD-organoids on regrowth day 7 after XAV-939 (10 μM), BSO (200 μM), auranofin (200 nM), harmine hydrochloride (10 μM), or BMH-21 (3 μM) treatment. *P < 0.05 vs. control group. Data are shown as the mean ± standard deviation. Independent samples *t* tests were performed for comparisons of the means between two groups, and Dunnett’s test was used to compare the means of multiple groups with that of the control group.

Based on the scRNA-seq results, characteristic signals of the non-cycling subpopulation were identified. To identify crucial signals for the survival of residual tumor cells and their transition to regrowth, chemical signaling inhibitors were tested in an in vitro model (Fig. 7A). When residual tumor cells were treated with the Wnt inhibitor XAV-939, the number of remaining cells was lower than that in the DMSO group. When F-actin was stained with phalloidin, numerous small ductal structures were observed around the CCD-organoids in the DMSO group, whereas a limited number of ductal structures were observed in the group treated with XAV-939. The PCR results also showed low expression of *MKI67*, indicating that regrowth was inhibited by XAV-939 treatment (Fig. 7B, C). With respect to ROS, BSO used for glutathione depletion and auranofin used for thioredoxin inhibition had no effect when used individually; however, when combined, they exerted a potent effect (Fig. 7D). Harmine, a dual-specificity tyrosine phosphorylation-regulated kinase inhibitor that broadly suppresses anti-ROS responses, induced cell death (Fig. 7E). Oligomycin and carbonyl cyanide 4-(trifluoromethoxy) phenylhydrazone, mitochondrial respiration inhibitors, did not induce cell death, whereas the RNA Pol I-inhibitor BMH-21 effectively killed residual tumor cells (Fig. 7E). Knockdown of *HMGCS2* in PLR123 cells had no impact on regrowth (**Fig. S8A–C**).

We determined whether the same mechanism could be observed in other colon cancer cell lines using LS174T cells, which are employed in colon cancer stem cell research. As with the PLR123 cells, a model was constructed where residual cells formed small duct structures after drug treatment and subsequently regrew (Fig. 7F, G). The trends in gene expression changes were similar (Fig. 7H). Inhibitor tests revealed that treatment with XAV-939, BSO + auranofin, or harmine inhibited regrowth, although a few residual cells were observed.

In contrast, treatment with BMH-21 completely killed the residual cells (Fig. 7I). These findings suggest Wnt, ROS, and RNA Pol I as potential therapeutic targets for CRC treatment targeting residual cancer cells and indicate that this model is a useful system for identifying therapeutically pathways and screening drugs.

### Evaluation of therapeutically important pathways in vivo

As no suitable in vivo model was available to analyze the process of cell progression to recurrence, we established an intravenous transplantation model using PLR123 cells engrafted in mouse lungs to form tumor nodules. In this model, residual cancer cells are efficiently induced after drug treatment, allowing for chemical inhibitor screening. The model forms small tumor nodules composed of undifferentiated cancer cells that frequently contain LGR5-positive cells for several weeks after transplantation [19]. Fourteen days after intravenous cell transplantation, small tumor nodules formed in the lungs. In the absence of drug treatment, the tumor nodules expanded due to the proliferation of Ki-67-positive tumor cells.

**Fig. 8:**
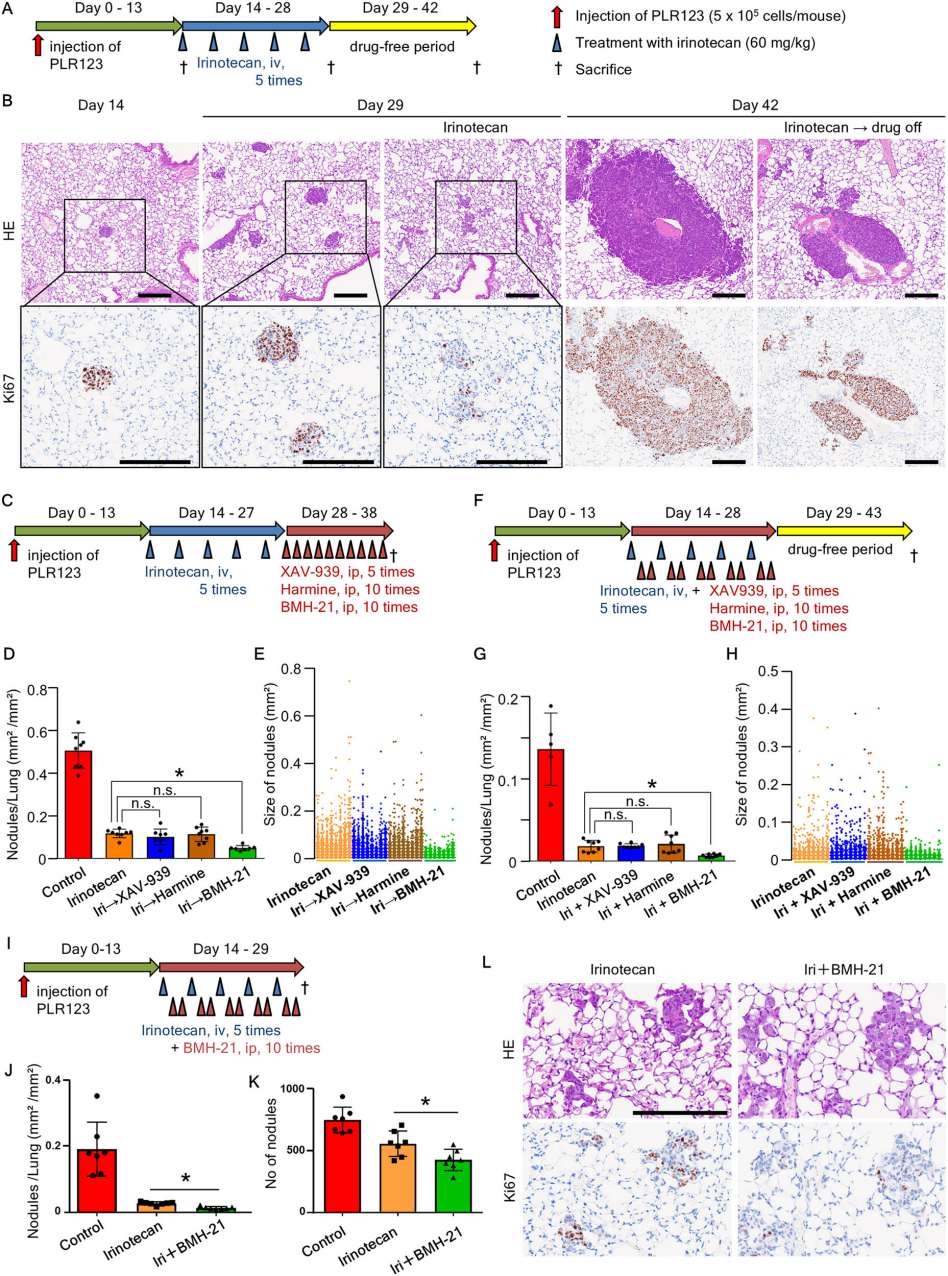
Sequence and cycle treatments in an intravenous transplantation model were established using PLR123 cells to evaluate chemical inhibitor effects. **A** Mouse lung intravenous transplantation model administered irinotecan (every 3 days). **B** Histopathology of lungs at 14, 29 (untreated and irinotecan-treated), and 42 (untreated and drug-free period) days following inoculation (HE, Ki67). Scale bars: 200 μm. **C**, **F**, **I** Design for sequential (**C**) and cycle (**F**, **I**) treatments. Chemical inhibitor treatments: in sequential treatments: XAV-939 (10 mg/kg, once every 2 days), harmine (20 mg/kg, daily), or BMH-21 (100 mg/kg, daily) and in cycle treatments: single irinotecan treatment and treatment with XAV-939 (10 mg/kg, once), harmine (20 mg/kg, twice), or BMH-21 (100 mg/kg, twice) were set as one cycle, and five cycle treatments were conducted. **D**, **E**, **G**, **H**, **J**, **K**) Image analysis of lung tumor nodules. **D**, **G**, **J** Total nodule area to the total lung tissue area and (**K**) total number of tumor nodules. Data are expressed as the mean ± standard deviation. Each dot represents an individual value. **E**, **H** Distribution of all individual tumor nodule sizes. **L** Histopathology of lungs on day 29 (HE, Ki67). Scale bar: 200 μm. *P < 0.05 vs. the group treated with irinotecan only.

On day 42 post-transplantation, duct formation was observed in the expanded nodules (Fig. 8A, B). After administering irinotecan five times on days 14, 17, 20, 23, and 26, small tumor nodules were observed on day 29 post-transplantation. Although Ki-67-positive cells remained present, the majority were Ki-67-negative non-cycling cells (Fig. 8A, B). On day 42 post-transplantation (after an irinotecan-free period), tumor nodule re-expansion due to resumed proliferation of Ki67-expressing tumor cells was observed (Fig. 8B). These results confirm that the recurrence process driven by residual cancer cells is reproduced in this model.

Based on the screening results of selected chemical inhibitors in vitro, we determined which phase is critical for residual cancer cell regrowth. To this end, we evaluated the effects of each chemical inhibitor on residual cancer cells in vivo using two treatment methods: sequential treatment, in which each inhibitor was administered after irinotecan treatment, and cycle treatment, in which each inhibitor was administered concurrently during treatment (Fig. 8C-H). In both experimental systems, no significant reduction in tumor size was observed for XAV-939 and harmine in image analysis of lung histological sections (Fig. 8D and G, Fig. S9A, B). By contrast, BMH-21 significantly decreased tumor size compared with irinotecan monotherapy in both experimental systems (Fig. 8D and G). Furthermore, analysis of size differences of individual tumor nodules in lung histological sections revealed that large tumor nodules were absent and small tumor nodules were predominant, indicating a reduction in the sizes of individual tumor nodules (Fig. 8E and H). In addition, tolerability was confirmed for the irinotecan and BMH-21 cycle treatment. Body weight decreased during the drug administration period by approximately 10% compared with the weight at the start of treatment and increased during the drug-free period (Fig. S9D). At the end of the drug-free period, no toxic changes were observed in the liver, spleen, kidneys, heart, lungs, jejunum, and colon on histopathological examination (Table S3).

To confirm the effects of cycle treatment with irinotecan and BMH-21 further, we observed the tumors after treatment ended on day 29 (Fig. 8I). BMH-21 significantly reduced the tumor size (Fig. 8J), and number of tumor nodules (Fig. 8K) compared with the effects of irinotecan monotherapy. Compared with irinotecan monotherapy, BMH-21 induced fewer Ki67-positive cells (Fig. 8L, Fig. S9E), whereas the number of cleaved caspase-3 positive cells was similar in the cells treated with irinotecan monotherapy and those treated with irinotecan and BMH-21 (Fig. S9F), indicating that BMH-21 treatment suppressed the transition of tumor cells from the non-cycling to the cycling state.

To confirm the inhibitory effect of BMH-21 in other cell lines, we evaluated its effect on LS174T cells. After cycle treatment with irinotecan and BMH-21, tumor growth was significantly reduced, similar to the findings in PLR123 cells (Fig. S10A–C). These experiments showing the significant inhibitory effect of BMH-21 treatment demonstrated that the phase in which protein synthesis was activated was critical for the regrowth of residual cancer cells.

## Discussion

In clinical cases where neoadjuvant therapy has been successful, despite nearly complete tumor disappearance, microscopic clusters composed of cancer cells with high stemness in extensive fibrotic regions have been observed, suggesting a potential source of recurrence. The residual cancer cells remaining after chemotherapy, therefore, must be characterized using a newly established model.

In this study, we established a three-dimensional culture model using PLR123 cells, known for their stable cultivation and the development of hierarchically organized cell populations. This model was designed to capture pathological morphological features, including common protein expression in residual cells after anticancer drug treatment, and represent the cellular structures and states we hypothesized as potential sources of recurrence. We demonstrated that residual cells exist in multiple states within the same tissue in actual clinical settings. Our model helped capture the state and characteristics of residual cancer cells reflecting drug-resistant conditions.

scRNA-seq is widely used as a useful tool for analyzing cell states; however, to conduct a detailed analysis of changes in cell states and cell populations over time, a fit-for-purpose reproducible experimental model is necessary. Using stable cell lines, we could observe changes over time and trajectories from scRNA-seq.

By conducting a time-course analysis of gene expression at the single-cell level during the regrowth process, the transition from non-cycling cells to cycling tumor cells could be elucidated. These trajectories included several key molecules. Although some of these have been reported previously, several new molecules were identified. Capturing the timing of regrowth has previously been difficult; however, our model enabled the identification of very limited cell populations or pathways involved in the timing of regrowth. Focusing on day 2, molecules that had not been previously highlighted were discovered. These are considered useful as datasets for the regrowth of residual cells. Furthermore, the regrowth model could be reproduced with commercially available cell lines, including LGR5-positive cells, and similar observations were made in in vitro and in vivo drug screening systems as with PLR123, indicating that the analysis of the regrowth process in highly stem-like colorectal cancer is useful.

To elucidate the potential of representative proteins in the regrowth phase, we examined in vitro and in vivo experiments. Inhibitor analysis showed that Wnt did not play a role in maintaining the non-cycling state. Therefore, although Wnt acts as a switch necessary to resume proliferation, inhibiting this signal is insufficient for depleting cancer cells. Various factors regulating ROS, garnering attention as a mechanism of anticancer drug resistance, were expressed in different phases. In the in vitro experiments, simultaneous suppression of multiple pathways, such as the glutathione and thioredoxin pathways, was required to eradicate residual cells. However, clear therapeutic effects were not achieved in the in vivo experiments using chemical inhibitors. Considering the findings of Mak et al. [21], the ROS control system can be inferred to have redundancy, with cancer cells developing sophisticated mechanisms to evade ROS stress. ROS-related molecules are thought to play an important role in maintaining residual cells.

In addition to clusters related to Wnt and ROS signaling and protein synthesis, a subpopulation including HMGCS2-positive cells was identified as a non-cycling subpopulation. HMGCS2 is a rate-limiting enzyme in ketone synthesis. The expression of UCP2, which is involved in mitochondrial respiration, was upregulated in the same cluster. The relationship between anticancer drug resistance and the switch of metabolism in cancer cells has been attracting attention [22]. In the present study, significant phenotypic changes were not observed upon HMGCS2 knockdown or treatment with mitochondrial respiration inhibitors. However, there are reports that HMGCS2 is involved in stemness maintenance in the mouse small intestine, suggesting that it may be prudent to continue monitoring its potential significance [23].

In the C4_day2_ cluster, protein synthesis was highly active. The subpopulation with transiently enhanced protein synthesis identified in our study was located upstream of Wnt-on cells in the RNA velocity plot. BMH-21, which inhibits RNA Pol I involvement in the transcription of ribosomal proteins, significantly suppressed the transition of tumor cells from the non-cycling to the cycling state both in vitro and in vivo. Protein synthesis is essential for the growth and survival of all cells. Although targeting protein synthesis as a cancer therapeutic strategy is already well-established, Pol I inhibitors have been investigated as cancer therapies [24, 25]. The present study provides new insights by demonstrating that this pathway is crucial in the early stages of regrowth of residual tumor cells, specifically before their transition to cycling. This finding highlights the potential of targeting protein synthesis pathways as an approach for complete eradication of residual tumor cells. Clara et al. [26] have reported that the degree of protein synthesis in ribosomes varies among CRC cells, with higher activity in subpopulations with high stemness. The genes characteristic of the C4_day2_ cluster were enriched for those related to protein synthesis; however, the role of each gene remains poorly understood, and further studies are needed to characterize their functions.

In conclusion, by linking the model with clinical residual cancer cell samples, this study provided a new method for evaluating the relapse process and a promising screening tool for drug discovery.

## Materials and methods

### Clinical patient samples

Primary CRC paraffin blocks were obtained from the archive of the Department of Surgery and Science, Kyushu University Hospital, Fukuoka, Japan. The samples were collected from patients who had undergone surgery between 1998 and 2013; all specimens had been embedded in paraffin after fixation in 10% neutral buffered formalin. Samples from 12 patients who had undergone chemotherapy prior to surgery were selected for the study (Table S1).

### Cell lines

In this study, two cell lines with high LGR5 expression (one derived from patient-derived xenograft, PDX and one commercially available cell line) were used. LGR5-positive colon cancer stem cells (PLR123, CHUGAI, Japan) were generated from PDX-PLR123 and cultured as described previously [18]. LS174T cells were purchased from the American Type Culture Collection (VA, USA). The cell line was selected after checking LGR5 expression among some cell lines. Details for culture conditions are provided in the Supplementary Materials and Methods file [27].

### CCD-organoid regrowth model

CCD-organoid cultures of PLR123 and LS174T were performed as previously described [20]. To observe regrowth after drug treatment, 300 nM 7-ethyl-10-hydroxycamptothecin (SN-38; Merck, NJ, USA) was added on day 10, and culturing continued until day 13. In constructing the regrowth model, simply switching the culture supernatant to a medium without anticancer agents allowed for regrowth, but the regrowth and its timing had limited reproducibility. Therefore, the Matrigel was dissolved with dispase I (Fujifilm Wako Pure Chemical Industries, Japan), and the CCD-organoids were carefully washed with Cellotion (Nippon Zenyaku Kogyo, Japan) to remove any residual chemical compounds. The CCD-organoids were then resuspended in Matrigel and dispensed into 96-well culture plates. The wells were overlaid with 200 μL of CCD-organoid culture medium.

In experiments using chemical inhibitors, L-buthionine sulfoximine (BSO; 200 μM; Merck) or auranofin (200 nM; Merck), harmine hydrochloride (200 μM; Selleck Biotech, Japan), oligomycin A (Merck), phenylhydrazone (FCCP; Merck), or BMH-21 (Selleck Biotech) was added. Details are provided in Supplementary Materials and Methods.

### Whole-mount immunostaining of CCD-organoids

CCD-organoids were collected by dissolving the Matrigel with dispase I, incubated in phosphate-buffered saline containing 4% paraformaldehyde (PFA) and 1% Triton-X100 on ice for 1 h, and then in blocking buffer (BlockAid Blocking Solution; Thermo Fisher Scientific, MA, USA) at room temperature for 1 h. Phalloidin-DyLight650 (Thermo Fisher Scientific) and 4′,6-diamidino-2-phenylindole (DAPI; Thermo Fisher Scientific) in blocking buffer were added, and incubation at room temperature was continued for 20 min. The CCD-organoids were then incubated in SeeDB2G Solution 1 (1/3× Omnipaque350 [Daiichi-Sankyo, Japan] with 2% saponin [Nacalai Tesque, Japan]), Solution 2 (1/2× Omnipaque350 with 2% saponin), and Solution 3 (1× Omnipaque350 with 2% saponin) at room temperature for 30 min each for optical clearing [28], and observed using a confocal fluorescence microscope (A1; Nikon, Japan) equipped with a 20× objective lens (CFI Plan Apochromat VC 20×; Nikon).

### Immunofluorescence for LGR5 and immunohistochemistry for CD44v9

Sections were subjected to immunohistochemistry staining with an anti-LGR5 antibody (clone 2U2E-2 [18, 29], CHUGAI, Japan). This anti-LGR5 antibody has been previously shown to be specific for LGR5 [18, 29] and does not cross-react with transfectant cells expressing LGR4 and LGR6, which have high homology with LGR5. Additionally, positive staining was observed sporadically in human crypt-base columnar cells (CBCs) and in the crypt base region of colorectal adenomas, further confirming its specificity for LGR5. The H-scores of LGR5 and CD44v9 expression were calculated for clinical samples. The H-score was calculated for each field as follows: H-score (0–300) = 0 × (% cells with negative expression) + 1 × (% cells with weak staining) + 2 × (% cells with moderate staining) + 3 × (% cells with strong staining). For LGR5, the H-score for each patient was calculated as the average H-score of the four or five fields.

### Bulk RNA-seq

Total RNA was converted to cDNA using the SMART-Seq v4 Ultra Low Input RNA Kit for Sequencing (Takara Bio, Shiga, Japan). Sequencing libraries were prepared using the Nextera XT DNA Library Prep kit (Illumina Inc., San Diego, CA, USA) and sequenced on a NextSeq500 system (Illumina). The RSEM software package (v1.2.6) [30] was used to map the RNA-seq data to the NCBI human genome assembly (GRCh38) and to measure transcript abundance (in fragments per kilobase of exon model per million mapped reads [FPKM]). Gene set enrichment analysis (GSEA) was performed using the GSEA software package v4.0.1. Hallmark gene sets were obtained from the MSigDB molecular signatures database v4.0[31]. Further information is given in the Supplementary Materials and Methods file.

### Single-cell RNA sequencing (scRNA-seq)

CCD-organoids were dissociated into single cells using TrypLE Select Enzyme (10×; Thermo Fisher Scientific), and large clusters were removed with a 20-μm cell strainer (pluriSelect GmbH, Leipzig, Germany). The viability of each sample (pretreatment, drug treated, regrowth day 2, day 4, day 6) was 99%, 95%, 86%, 94%, and 93%, respectively. Single-cell libraries were generated using the GemCode Single-Cell Instrument and Chromium Single Cell 3′ Library, Gel Bead Kit v2 (10× Genomics, Pleasanton, CA, USA) according to the manufacturer’s protocol.

Sequencing libraries were generated with unique sample indices and sequenced on a HiSeq 2500 instrument using HiSeq PE Rapid Cluster Kit v2 (Illumina).

### scRNA-seq data analysis

The sequencing data were processed using the Cell Ranger Single Cell Software Suite 1.3.1 (10× Genomics). This output was imported into SCANPY (v1.4) [32] for quality control and downstream analysis. In the quality control step, we filtered out genes detected in fewer than three cells and in cells in which <200 genes had nonzero counts and >10% of the counts belonged to mitochondrial genes. Clustering was performed using the neighbors and Louvain tools (v0.6.1) [33] in SCANPY; the cells were visualized using UMAP [34].

To assess the differences in composition throughout the regrowth process, we performed a global clustering analysis. First, we combined the unique molecular identifier count and filtered out low-quality cells. After applying these criteria, 20,307 cells and 19,338 genes were retained and normalized. The batch effect was corrected by matching mutual nearest neighbors using BBKNN (v1.3.2) [35]. Differentially expressed genes in each module were ranked using the rank_genes_groups tool and compared among modules. Gene ontologies of the top 100 differentially expressed genes in each module were assigned using the DAVID functional annotation tool (v6.8, http://david.ncifcrf.gov/) [36] and Metascape (https://metascape.org) [37].

For RNA velocity analysis, the data were converted to spliced and unspliced reads using the velocyto package (v0.17.17) in SCANPY. To infer the directionality of cell transition during the regrowth phase, we estimated RNA velocity [38] using the stochastic model in scVelo (v0.1.16; https://github.com/theislab/scvelo). The velocity graph was used to project the velocities into the UMAP [39] embedding as a stream plot. Further information is given in Supplementary Materials and Methods.

### Histopathological analysis of CCD-organoid samples and intravenous transplantation model mice

CCD-organoid samples were collected by dissolving the Matrigel in Dispase I. Following this, the samples were fixed in 4% PFA at room temperature for 30 min and centrifuged in an unsolidified agarose solution. The samples were then processed and embedded in paraffin using the AMeX method [40]. Lungs from intravenous transplantation model mice were fixed in 10% neutral buffered formalin. The samples were embedded in paraffin according to the standard method. The paraffin blocks were sectioned (to 4 μm) and stained with HE or periodic acid–Schiff–Alcian Blue using standard methods. Two certified pathologists (MS and MY) performed the histological analysis.

### Image analysis

The HALO AI image analysis software (v3.2; Indica Labs, NM, USA) was used to analyze tumor nodules in lung tissues. Using the “classifier” function, a pathologist (MY) annotated “tumor nodule,” “lung tissue,” and “background” in immunohistochemistry slides stained for human mitochondria. The classified results were output after iterative training by the machine. Two pathologists (MS and MY) validated the tissue classification results.

### Intravenous transplantation model establishment and preparation of lungs, including tumor nodules

NOG mice were used to establish an intravenous transplantation model by grafting tumor cells in the lungs. Male NOG mice were purchased from CLEA Japan and used in experiments at approximately 8 weeks of age. PLR123 cells were suspended in PBS, and 5 × 10^5^ cells/200 μL were injected intravenously into the tail vein of an NOG mouse. Tumor-bearing mice were used for subsequent studies. Mice injected with PLR123 cells received irinotecan treatment, followed by a drug-free period and lung sampling at various points. A sequential treatment experiment evaluated the effects of irinotecan combined with chemical inhibitors (XAV-939: Selleck Chemicals LLC., TX, USA, harmine: FUJIFILM Wako Pure Chemicals Co., Japan, or BMH-21: Selleck Chemicals LLC., TX, USA). The dose and frequency of administration of each chemical inhibitor were determined as previously described in the literature (XAV-939 [41], harmine [42], BMH-21 [26]). Lung samples were collected 38 days post-injection after sacrificing the mice by exsanguination. The details of the method are shown in the Supplementary Materials and Methods file.

### Quantification and statistical analysis

Data are shown as mean ± standard deviation. Statistical analyses were performed using the GraphPad Prism v7.04 (GraphPad Software, San Diego, CA, USA) and JMP software v17.2.0 (SAS Institute, Cary, NC, USA). For the in vitro evaluations, independent samples *t*-tests were performed for comparisons of the means between two groups, and Dunnett’s test was used to compare the means of multiple groups with that of the control group. In image analysis, one-way analysis of variance followed by Tukey’s post-hoc test or an independent samples *t*-test was used to assess statistical significance. Statistical significance was set at *P* < 0.05.

### Ethics approval and consent to participate

All methods were performed in accordance with the relevant guidelines and regulations. This study with clinical samples was approved by the Institutional Review Board of Kyushu University (Approval No. 27-280). All patients provided written informed consent prior to surgery for the collection and banking of biological specimens, including DNA and RNA, for potential use in future genetic analyses and related research. For each specific study utilizing these samples, information regarding the study objectives, methods, and patient rights, including the right to refuse participation (opt-out), was publicly disclosed on the institutional website. Patients were provided the opportunity to decline participation through the opt-out procedure.

All animal experiments were approved by the Institutional Animal Care and Use Committee of the Central Institute for Experimental Animals (Approval No. 22006 A).

## Supplementary information

Supplementary materials

Supplemental Figures

Table S1. Information on the 12 cllinical cases

Table S1. Cluster gene lists

Table S3. Histopathological evaluation

## Data Availability

The data generated in this study are available upon request from the corresponding author.
