# Supplementary material for: Colorectal cancer cell line-derived organoid model with stem cell properties captures the regrowing state of residual cancer cells after neoadjuvant chemotherapy
Source: Cell Death Discov. 2025 Jun 20;11:282. doi: 10.1038/s41420-025-02567-w (PMC12179298; doi:10.1038/s41420-025-02567-w)
Supplement: Supplementary file 1 — Supplementary materials [file 41420_2025_2567_MOESM1_ESM.docx]

**Colorectal cancer organoid model with stem cell properties captures the regrowing state of residual cancer cells after neoadjuvant chemotherapy**

Kiyotaka Nakano,^1,#,*^ Eiji Oki,^2,#^ Masaki Yamazaki,^1,#^ Masami Suzuki,^3^ Shigeto Kawai,^1^ Takanori Fujita,^4^ Atsuhiko Kato,^1^ Yoko Zaitsu,^2^ Tomoko Jogo,^2^ Chie Kato,^1^ Takeshi Watanabe,^5^ Eri Hashimoto,^1^ Chiyoko Nishime,^3^ Etsuko Fujii,^1^ Koji Ando,^2^ Genta Nagae,^4^ Norifumi Harimoto,^2^ Mitsuhiko Ota,^2^ Hiroshi Saeki,^2^ Hiroyuki Aburatani,^4^ Yoshihiko Maehara,^2,6^ and Tatsumi Yamazaki^3,7^

^1^Translational Research Division, Chugai Pharmaceutical Co., Ltd., Chugai Life Science Park Yokohama 216 Totsuka-cho, Totsuka-ku, Yokohama, Kanagawa 244-8602 Japan

^2^Department of Surgery and Science, Graduate School of Medical Sciences, Kyushu University, 3-1-1 Maidashi, Higashi-ku, Fukuoka-shi, Fukuoka, 812-8582 Japan

^3^Central Institute for Experimental Medical and Life Science, 3-25-12 Tonomachi, Kawasaki-ku, Kawasaki, 210-0821, Japan

^4^Genome Science Division, Research Center for Advanced Science and Technology, The University of Tokyo, 4-6-1 Komaba, Meguro-ku, Tokyo 153-8904 Japan

^5^Chugai Research Institute for Medical Science, Inc., Chugai Life Science Park Yokohama 216 Totsuka-cho, Totsuka-ku, Yokohama, Kanagawa 244-8602 Japan

^6^Kyushu Central Hospital of the Mutual Aid Association of Public School Teachers, 3-23-1 Shiobaru, Minami-ku, Fukuoka-shi, Fukuoka 815-8588 Japan

^7^Chugai Pharmaceutical Co., Ltd., 1-1 Nihonbashi-Muromachi 2-chome, Chuo-ku, Tokyo 103-8324 Japan

# These authors contributed equally.

**^*^Corresponding author:**

Kiyotaka Nakano

Translational Research Division, Chugai Pharmaceutical Co., Ltd., Chugai Life Science Park Yokohama 216 Totsuka-cho, Totsuka-ku, Yokohama, Kanagawa 244-8602 Japan

Tel.: +81-45-870-3351

Fax: +81-45-870-3367

Email: [nakanokyt@chugai-pharm.co.jp](mailto:nakanokyt@chugai-pharm.co.jp)

**Supplementary Material and Methods**

**PLR123 cell culture**

PLR123 cells were cultured in Dulbecco’s modified Eagle’s medium (DMEM)/F12 supplemented with 1× antibiotic–antimycotic, 1× N-2 Supplement, 4 mg/mL AlbuMAX I Lipid-Rich BSA, 20 ng/mL human EGF, 20 μg/mL human insulin (all from Thermo Fisher Scientific, Waltham, MA, USA), 2.9 mg/mL glucose (Merck, Rahway, NJ, USA; total 6.0 mg/mL glucose), 4 μg/mL heparin (Merck), and 10 ng/mL human FGF2 (Reprocell, Yokohama, Kanagawa, Japan). To knock down *HMGCS2* in PLR123 cells, TRC lentiviral shRNA targeting human *HMGCS2* (TRCN0000045859) was obtained from Merck. PLR123 cells were infected with lentivirus particles in the presence of 8 μg/mL polybrene and screened with puromycin (Thermo Fisher Scientific) to establish stable cell lines.

**Culture for organoid regrowth model**

The cells were suspended in Matrigel (Corning, NY, USA) and seeded in 24-well culture plates (250 cells/50 μL Matrigel droplet/well). After solidification of the Matrigel at 37 °C, 650 μL organoid culture medium comprising Advanced DMEM/F12 medium supplemented with penicillin/streptomycin, 10 mM HEPES, 2 mM GlutaMAX, 1× B-27 Supplement, 1× N-2 Supplement (Thermo Fisher Scientific), and 1 mM N-acetylcysteine (Merck) was added. The cells were cultured at 37 °C in the presence of 5% CO_2_. For SN-38 treatment of the organoids, 300 nM 7-ethyl-10-hydroxycamptothecin (SN-38; Merck) was added on day 10, and the culture was continued until day 13.

In experiments using chemical inhibitors, l-buthionine sulfoximine (BSO; 200 μM; Merck) or auranofin (200 nM; Merck), harmine hydrochloride (200 μM Selleck Biotech), oligomycin A (Merck), phenylhydrazone (Merck), or BMH-21 (Selleck Biotech) was added. Serial microscopic observation was conducted using a Confocal Quantitative Image Cytometer CQ1 (Yokogawa Electric Corporation, Tokyo, Japan) equipped with a 10× objective lens (UPLSAPO10×2, Olympus, Tokyo, Japan). The CellTiter-Glo 3D Cell Viability Assay (Promega, WI, USA) was used to evaluate organoid growth. Luminescence was measured using a plate reader (EnSpire 2300; PerkinElmer, MA, USA).

**Immunofluorescence for LGR5**

In brief, after incubation with the primary antibody (2.5 μg/mL), the sections were incubated with a secondary antibody conjugated with biotin (5.0 μg/mL, ab98784; Abcam, Cambridge, UK), and the reaction was visualized with Qdot 605 Streptavidin (diluted 1: Q10101MP; Thermo Fisher Scientific). Alternatively, after incubation with the primary antibody (1.0 μg/mL), the sections were incubated with a secondary antibody conjugated with polymer-horseradish peroxidase (K4001, ready-to-use; DAKO, Glostrup, Denmark), and the reaction was visualized with Alexa Fluor 488-labeled tyramide (diluted 1:100, T20912; Thermo Fisher Scientific).

**H-score for LGR5 and CD44v9**

For LGR5, we randomly selected four or five fields per slide stained for LGR5 expression for each patient sample. For CD44v9, we performed staining under a bright field microscope, subjecting the entire tissue to analysis. The intensity and area of expression were assessed by certified pathologists. Staining intensity was classified as weak, moderate, or strong, and the area for each intensity grade was estimated as a percentage of the area of the entire field.

**Immunohistochemistry**

Sections were probed with the following primary antibodies: anti-CD44v9 (1 μg/mL, RV3, #LKG-M001, CosmoBio, Tokyo, Japan), HMGCS2 (0.5 μg/mL, PA5-55620; Thermo Fisher Scientific), anti-Ki67 (SP6, 50×, ab16667; Abcam, or MIB-1, 1–5 μg/mL, M7240; Agilent, Santa Clara, CA, USA), anti-villin (1.0 μg/mL, M3637; Agilent), anti-p21 (0.2 μg/mL, 2947; Cell Signaling Technology, Beverly, MA, USA), anti-human mitochondria (2,000×, 113-1; Merck Millipore, Darmstadt, Germany), anti-E-cadherin (0.5 μg/mL, ab40772, Abcam), anti-Vimentin (×100, SP20, 413551, Nichirei, Japan), anti-ZEB1 (×200, ab87280, Abcam), anti-nuclei antibody (×500, 3E1.3, MAB4383, Sigma-Aldrich, Saint Louis, MO) and anti-cleaved caspase-3 (×1500, Asp175, #9661, Cell Signaling Technology). For immunofluorescence, after incubation with the primary antibodies, the sections were incubated with an anti-mouse (5.0 μg/mL, A90-238B; Bethyl Laboratories, Montgomery, TX, USA) or anti-rabbit biotinylated IgG (5.0 μg/mL, BA-1000; Vector Laboratories, Burlingame, CA, USA) secondary antibody, and the reaction was visualized with Alexa Fluor 568-labeled streptavidin (5.0 μg/mL, S11226; Thermo Fisher Scientific). All the specimens were stained with a Qnuclear Deep Red stain (Q10363; Thermo Fisher Scientific) or DAPI (62248; Thermo Fisher Scientific) to detect nuclei. The specimens were observed using a confocal microscope (C1 or A1; Nikon, Tokyo, Japan). Image data were processed using the NIS-Elements software (Nikon). Immunohistochemistry was performed using EnVision reagent (K4001 or K4003; Agilent) or BOND Polymer Refine Detection (DS9800; Leica, Wetzlar, Germany) or using the labeled streptavidin biotinylated antibody method with biotinylated anti-rat IgG (5.0 μg/mL, A100-322B; Bethyl Laboratories) or biotinylated anti-goat IgG (5.0 μg/mL, BA-5000; Vector Laboratories) as the secondary antibody. The immunoreaction was visualized using streptavidin-labeled horseradish peroxidase (SA-5704; Vector Laboratories) or a peroxidase-diaminobenzidine reaction. The sections were counterstained with hematoxylin.

Quantitative analysis of Ki-67 positive human tumor cells was performed in the experimental groups shown in Fig. 8L. The left lung lobe specimens from the first four cases in each of the Day 29 irinotecan-treated and Iri+BMH-21-treated groups were analyzed. After immunohistochemistry (IHC) by staining for human cell nuclei to recognize human tumor cells and Ki-67 IHC to identify human proliferating cells, positive cells were identified using the HALO AI system and the percentage of Ki-67 positive cells among human tumor cells was calculated by dividing the number of Ki-67 positive cells by the number of human nucleus-positive cells.

**Animal studies**

To characterize the intravenous transplantation model treated with a cytotoxic anticancer agent, mice were intravenously injected every 3 days for 2 weeks (five times in total) with 60 mg/kg irinotecan (Daiichi-Sankyo, Tokyo, Japan) from day 14 after injection of PLR123 cells. After irinotecan administration, a 14-day drug-free period was included. An irinotecan-untreated group was included as a control. All animals were exsanguinated under inhalation anesthesia with isoflurane. At necropsy, lungs were sampled from animals at 14 (untreated), 29 (untreated and irinotecan-treated), and 42 (untreated and drug-free period after the irinotecan treatment) days after the injection of PLR123 cells (n = 4/group).

The effects of chemical inhibitors were evaluated in two treatment experiments. In the sequential treatment experiment, 14 days after the injection of PLR123 cells, irinotecan (60 mg/kg) was administered five times intravenously. Subsequently, XAV-939 (intraperitoneal doses of 10 mg/kg, administered once every 2 days for five doses), harmine (daily intraperitoneal doses of 20 mg/kg for 10 days), or BMH-21 (daily intraperitoneal doses of 100 mg/kg for 10 days) was administered.

In the cycle treatment study, a single irinotecan treatment (60 mg/kg, intravenously) and treatment with XAV-939 (one intraperitoneal dose of 10 mg/kg), harmine (two intraperitoneal doses of 20 mg/kg), or BMH-21 (two intraperitoneal doses of 100 mg/kg) were set as one cycle. Five cycle treatments were conducted 14 days after injection of PLR123 cells, followed by a 14-day drug-free period. Lungs were sampled after sacrifice by exsanguination at 42 days (untreated control n = 5, irinotecan and chemical inhibitors n = 8) after injection of PLR123 cells. In addition, the liver, spleen, kidneys, heart, jejunum, and colon from control, irinotecan alone, and irinotecan and BMH-21 groups were sampled to assess tolerability.

To confirm the effects of cycle treatment of irinotecan and BMH-21, a single irinotecan treatment (60 mg/kg, intravenously) and BMH-21 treatment (two intraperitoneal doses of 100 mg/kg) were set as one cycle. Five cycle treatments were conducted 14 days after the injection of PLR123 cells. Lungs were sampled after exsanguination 29 days after the injection of PLR123 cells (end of cycle treatment; n = 7/group).

To analyze the effects of cycle treatment in LS174T cells, LS174T cells were injected into the tail vein (1 × 10^6^ cells/mouse). A single irinotecan treatment (60 mg/kg, intravenously) and BMH-21 treatment (two intraperitoneal doses of 100 mg/kg) were set as one cycle, and five cycle treatments were conducted 14 days the injection of LS174T cells, followed by a 14-day drug-free period. Lungs were sampled after sacrifice by exsanguination at 42 days (n = 5/group) after injection of LS174T cells. Animals in the untreated control group were sacrificed on day 27 because of the deterioration of their general condition due to tumor masses in their systemic organs (particularly in subcutaneous brown fat and systemic lymphoid tissues).
