# Supplementary material for: Colorectal cancer cell line-derived organoid model with stem cell properties captures the regrowing state of residual cancer cells after neoadjuvant chemotherapy
Source: Cell Death Discov. 2025 Jun 20;11:282. doi: 10.1038/s41420-025-02567-w (PMC12179298; doi:10.1038/s41420-025-02567-w)
Supplement: Supplementary file 2 — Supplemental Figures [file 41420_2025_2567_MOESM2_ESM.pdf]

Fig. S1

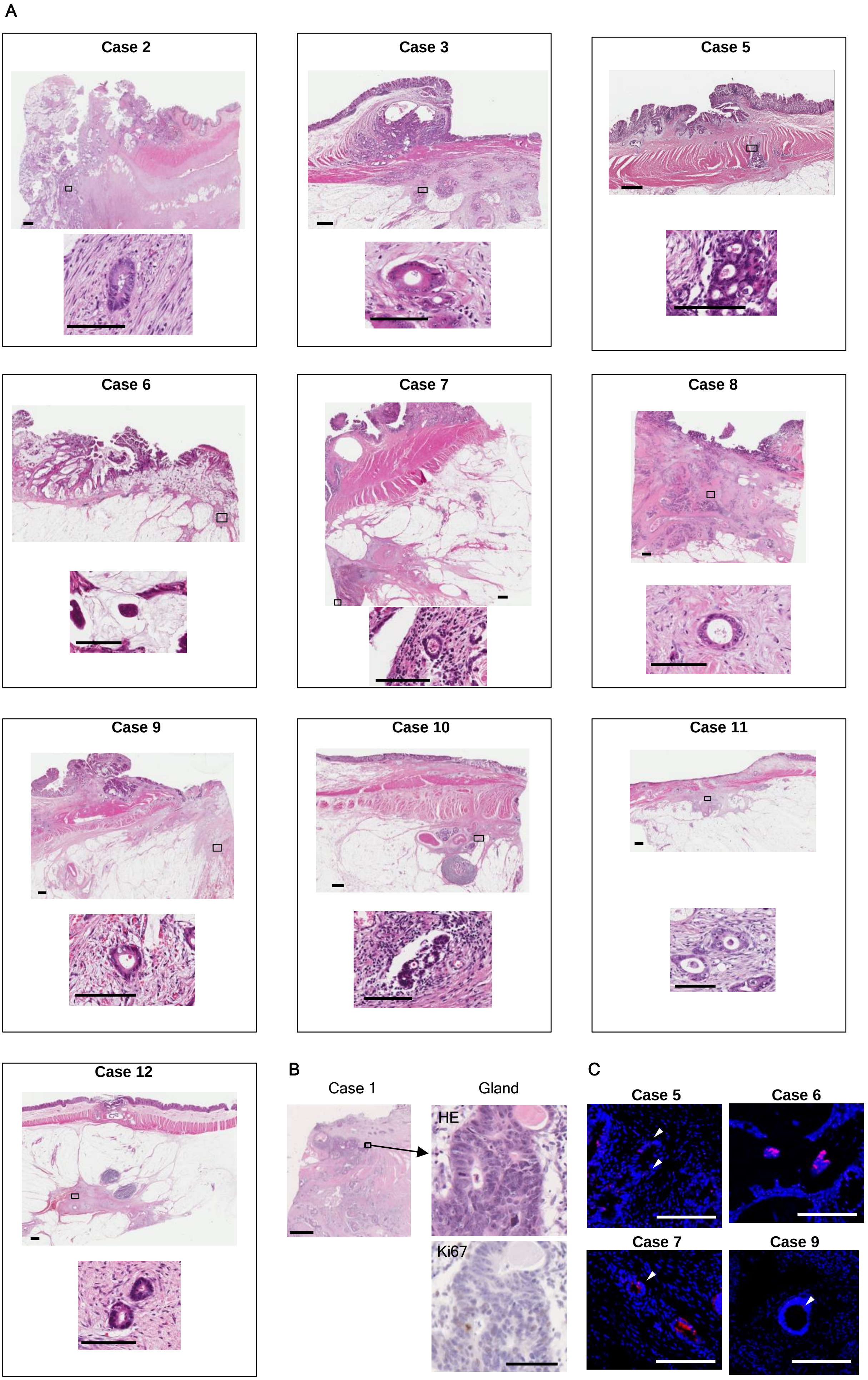
A
Case 2
Case 3
Case 5
Case 6
Case 7
Case 8
Case 9
Case 10
Case 11
Case 12
B
Case 1
Gland
HE
Ki67
C
Case 5
Case 6
Case 7
Case 9

Figure S1. Location and structure of small clusters in each patient (12 cases)

(A) Histopathological image for 12 CRC cases. in each case, the black rectangle in the low-magnification image of the sample (upper image) indicates the location of the high-magnification image in which the small cluster is displayed (lower image). HE staining. Scale bars, 1 mm for upper images and 100 μm for lower images. (B) The expression of Ki67 in glandular structure in Case 1. Scale bar, 100 μm. (C) The expression of LGR5 in small cluster in Case 5, 6, 7 and 9. Arrow heads indicated the expression of LGR5. Scale bar, 100 μm.

Fig. S2

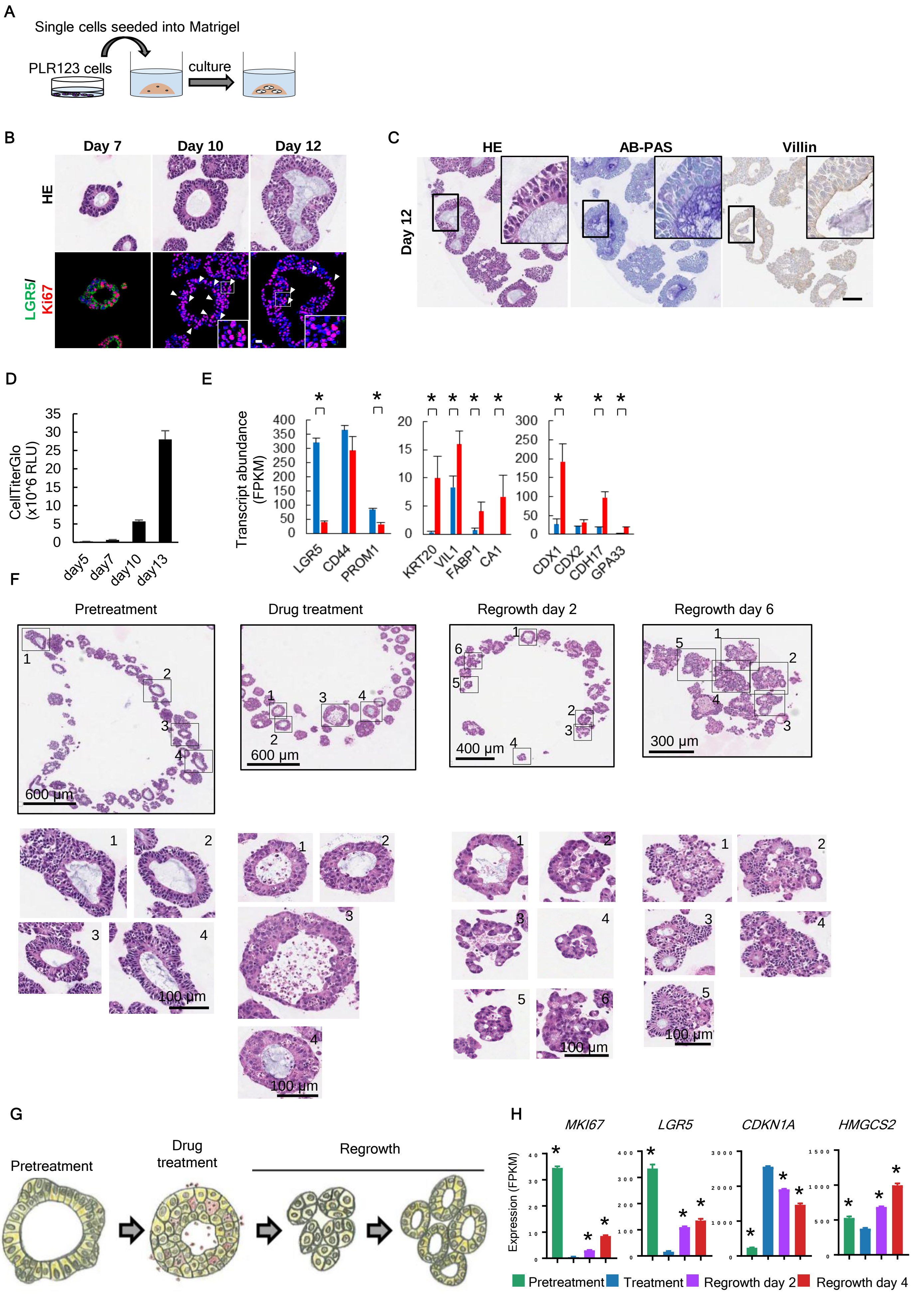

A
Single cells seeded into Matrigel
PLR123 cells
culture
B
Day 7
Day 10
Day 12
HE
LGR5/
Ki67
C
HE
AB-PAS
Villin
Day 12
D
CellTiterGlo
(x10^6 RLU)
day5
day7
day10
day13
E
Transcript abundance
(FPKM)
LGR5
CD44
PROM1
KRT20
VIL1
FABP1
CA1
CDX1
CDX2
CDH17
GPA33
F
Pretreatment
Drug treatment
Regrowth day 2
Regrowth day 6
600 µm
600 µm
400 µm
300 µm
100 µm
G
Pretreatment
Drug
treatment
Regrowth
H
MKI67
LGR5
CDKN1A
HMGCS2
Expression (FPKM)
Pretreatment
Treatment
Regrowth day 2
Regrowth day 4

Figure S2. Characterization of PLR123 derived cancer organoid.

(A) Experimental design for producing organoids. (B) Histopathological images of cancer organoids established from PLR123 cells. Arrowheads indicate LGR5-positive cells. Green, LGR5; red, Ki67; blue, DAPI. Inset, high magnification for LGR5-positive cells. Scale bar: 20 μm. (C) Histopathological images of PLR123 organoids on day 12 stained with periodic acid-Schiff-Alcian blue and for villin. Scale bars: 100 μm. (D) Cell viability in organoids over time (n=3). (E) Gene expression analysis for stem cell markers (*LGR5*, *CD44*, *PROM1*), differentiation markers (*KRT20*, *VIL1*, *FABP1*, *CA1*), and intestinal differentiation markers (*CDX1*, *CDX2*, *CDH17*, *GPA33*) for 2D culture (blue) vs. organoid (Day 10) (red). (F) Time-course images acquired during organoid regrowth after SN-38 treatment. The representative images shown in Fig. 3C were selected from these samples. Boxes in the images on the left at low magnification indicate the locations (numbered) of the images on the right. The scale is indicated in each image. (G)Schematic image of regrowth process of organoids after treatment with SN-38. (H) Changes in gene expression of *LGR5*, *MKI67*, *CDKN1A*, and *HMGCS2* after anticancer drug treatment and washout in 2D cultured parent PLR123 cells were analyzed. *P < 0.05 vs. treatment control group. Data are shown as mean ± standard deviation.

Fig. S3

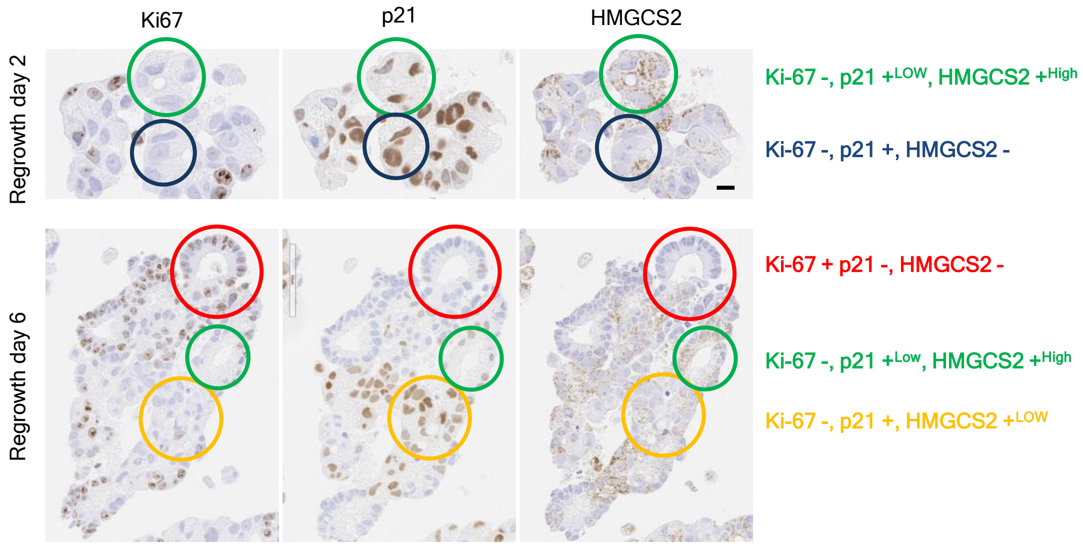

Figure S3. Expression of Ki67, p21, and HMGCS2 in the regrowth phase after treatment with SN-38

Expression of Ki67, p21 and HMGSC2 at regrowth-D2 and -D6 in the organoid model. Yellow circle indicated Ki67-,p21+,HMGCS2$^{+/low}$. Green circle indicated Ki67-,p21$^{+/low}$, HMGCS2$^{+/high}$. Red circle indicated Ki67+,p21-,HMGCS2-. This area showed small cluster-cuboid structure. Scale bar, 10 μm.

Fig. S4

Case 8 HMGCS2

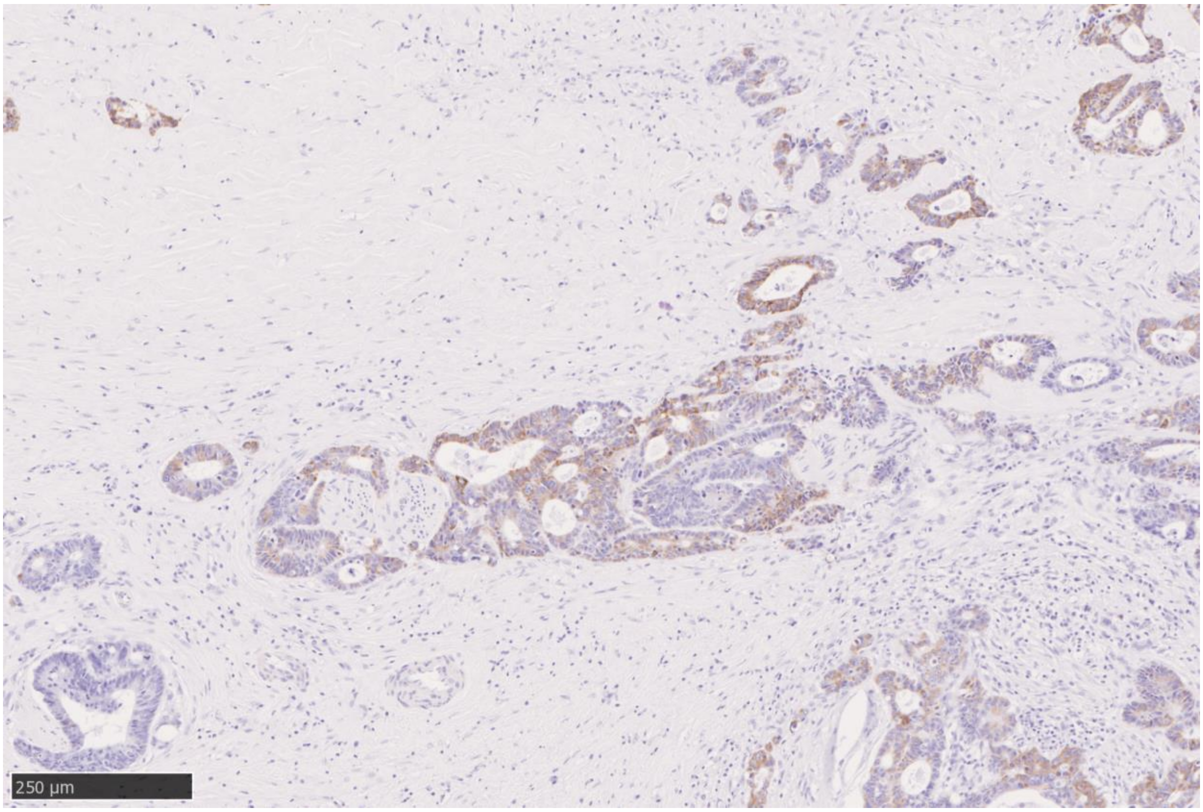

Figure S4. Low magnification image for HMGCS2 in Case 8

IHC image of HMGCS2 in Case 8. HMGCS2 was diffusely expressed in small clusters. Furthermore, it was also diffusely expressed in glandular structures. Scale bar, 250 μm.

Fig. S5

A

Sample	Number of cells	Median UMI counts /cell	Median genes /cell
Pretreatment	2,754	27,253	3,842
Drug treatment	2,946	3,467	941
Regrowth day 2	1,698	12,014	2,558
Regrowth day 4	4,174	3,272	916
Regrowth day 6	3,572	25,728	3,637

B

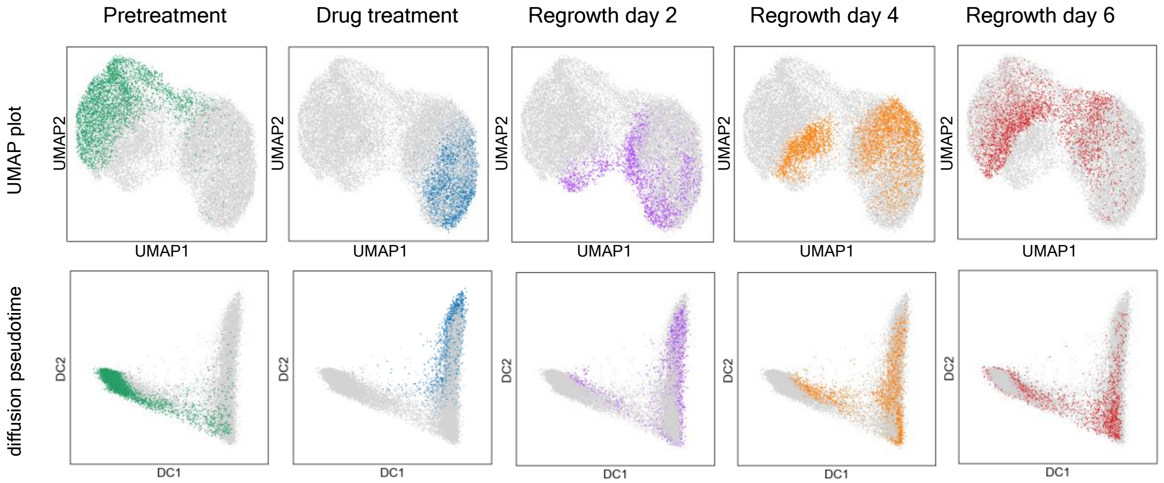

C

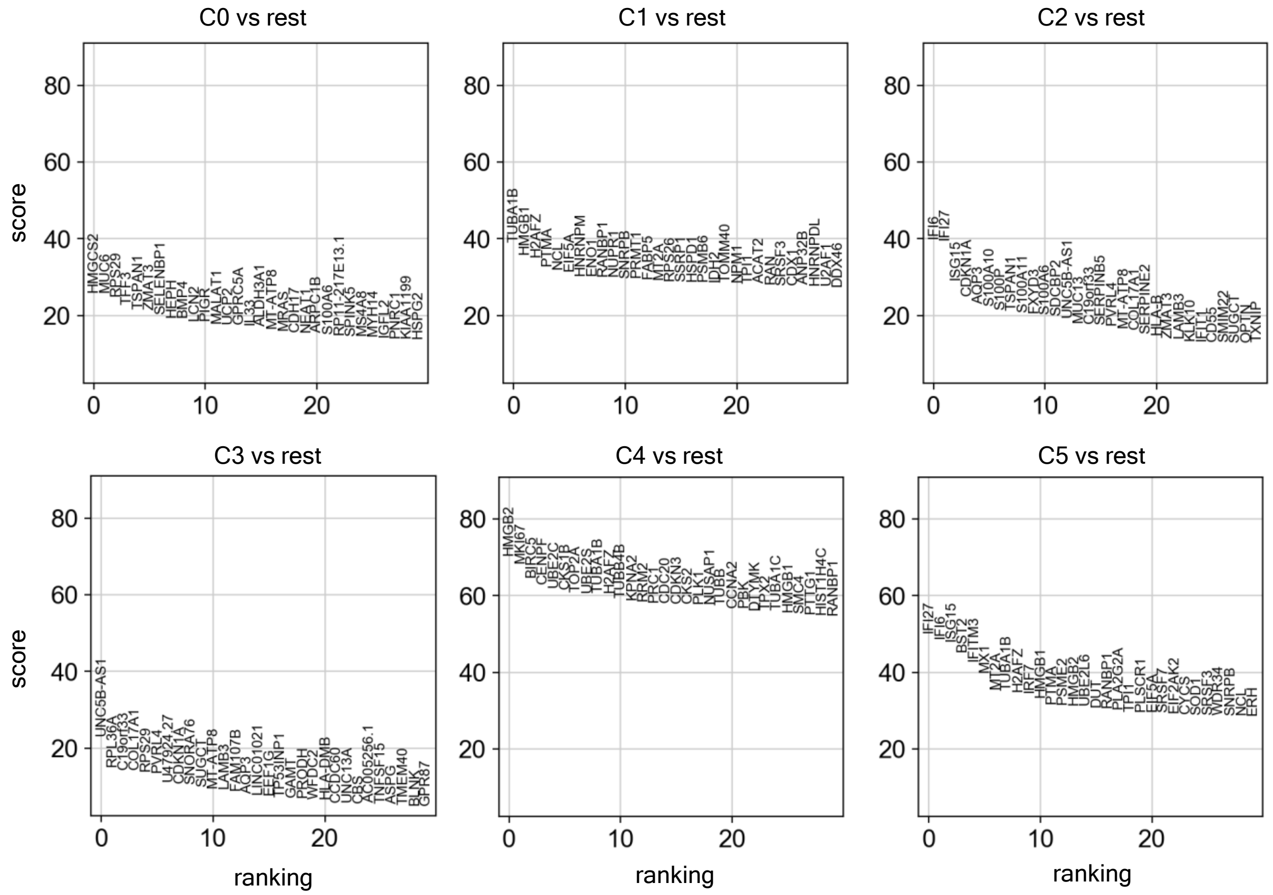

Figure S5. scRNA-seq analysis of PLR123 organoids.

(A) Sequencing metrics of an individual library sequenced using HiSeq 2500. **(B)** UMAP and diffusion pseudotime visualization of 15,114 cells from PLR123 organoids. Cells are colored by sample at each time point. **(C)** Ranking of highly differentially expressed genes in each cluster of PLR123 organoid cells, including pretreated and drug-treated cells and cells on regrowth days 2, 4, and 6.

Fig. S6

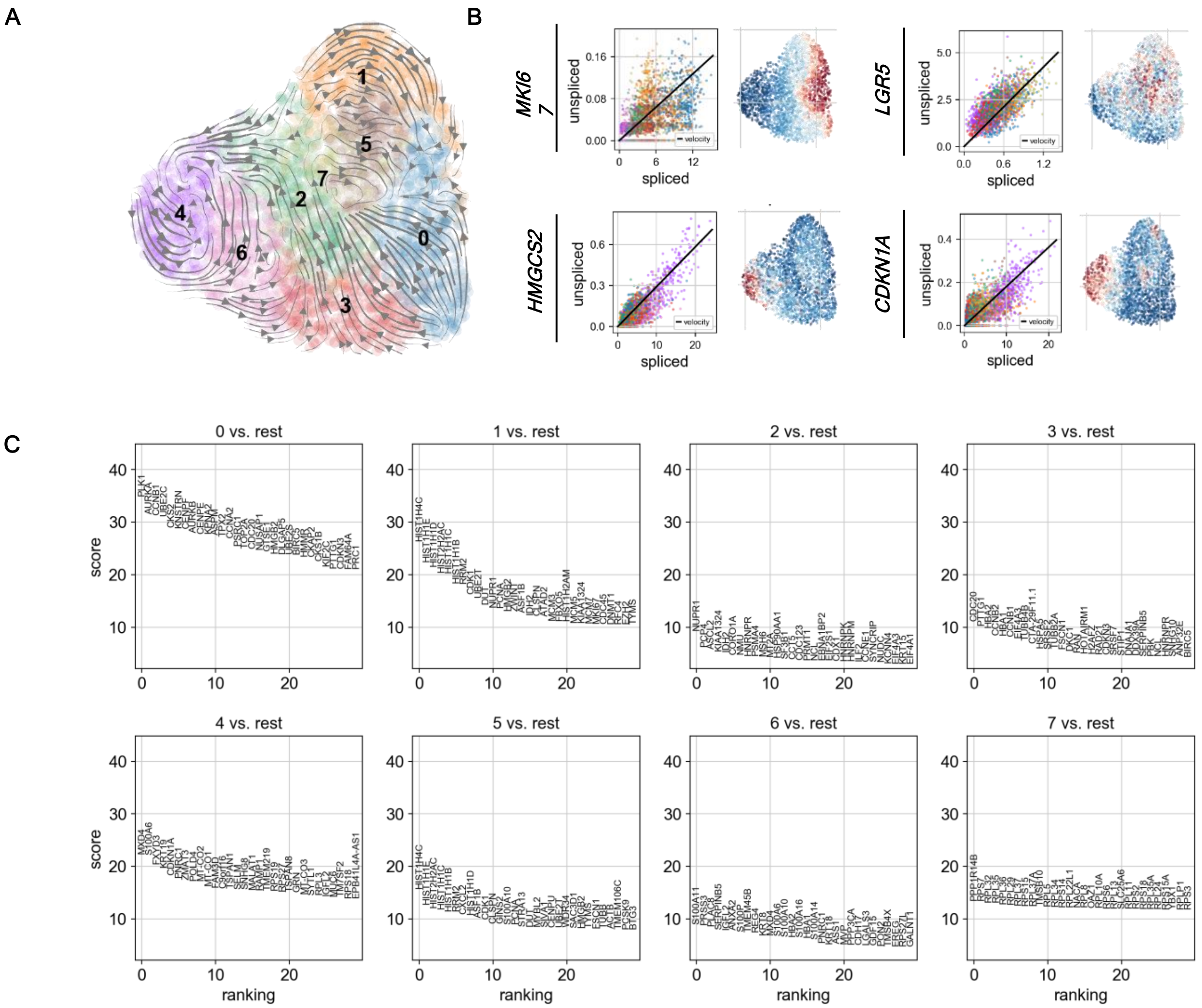

Figure S6. scRNA-seq analysis of PLR123 organoids.

(A) UMAP visualization of 2,754 cells on pretreatment sample. **(B)** Summary of RNA velocity analysis of the indicated genes. **(C)** Ranking of highly differentially expressed genes in each cluster.

Fig. S7

A

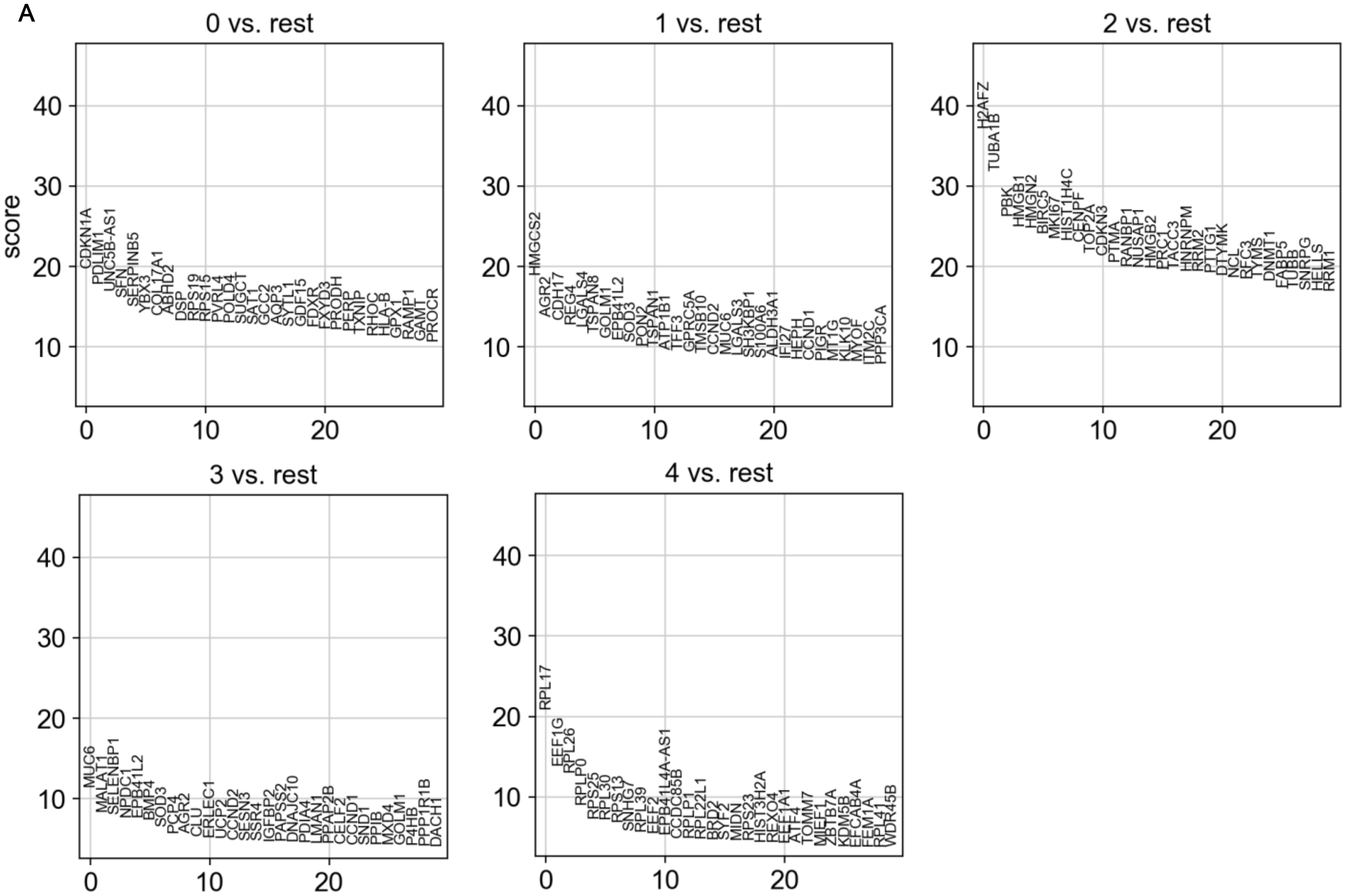

B

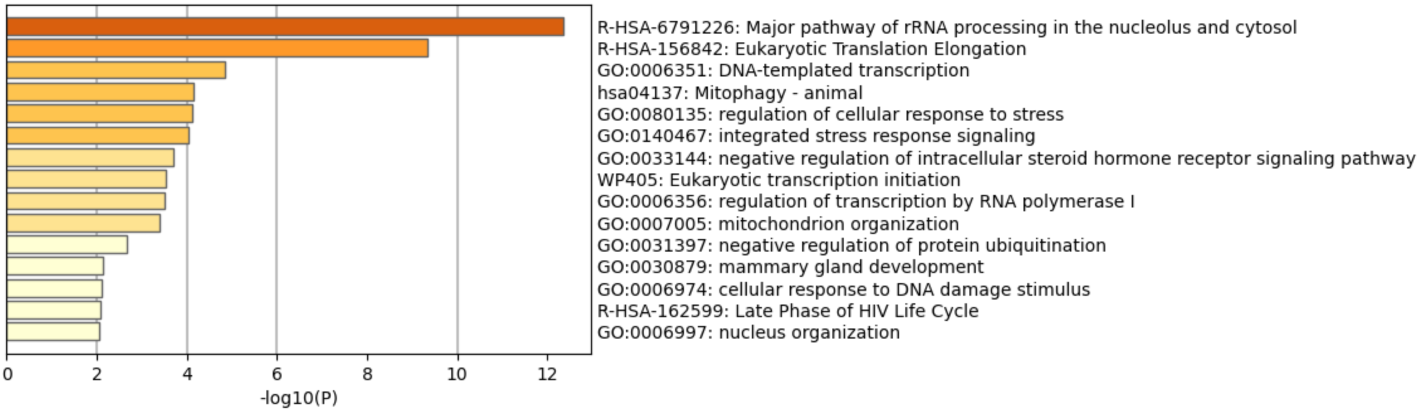

Figure S7. ScRNA-seq analysis of PLR123 organoid regrowth day 2. (A) Ranking of highly differentially expressed genes in each cluster of PLR123 organoid cells on regrowth day 2. **(B)** Pathway analysis of $C4_{day2}$ using Metascape.

Fig. S8

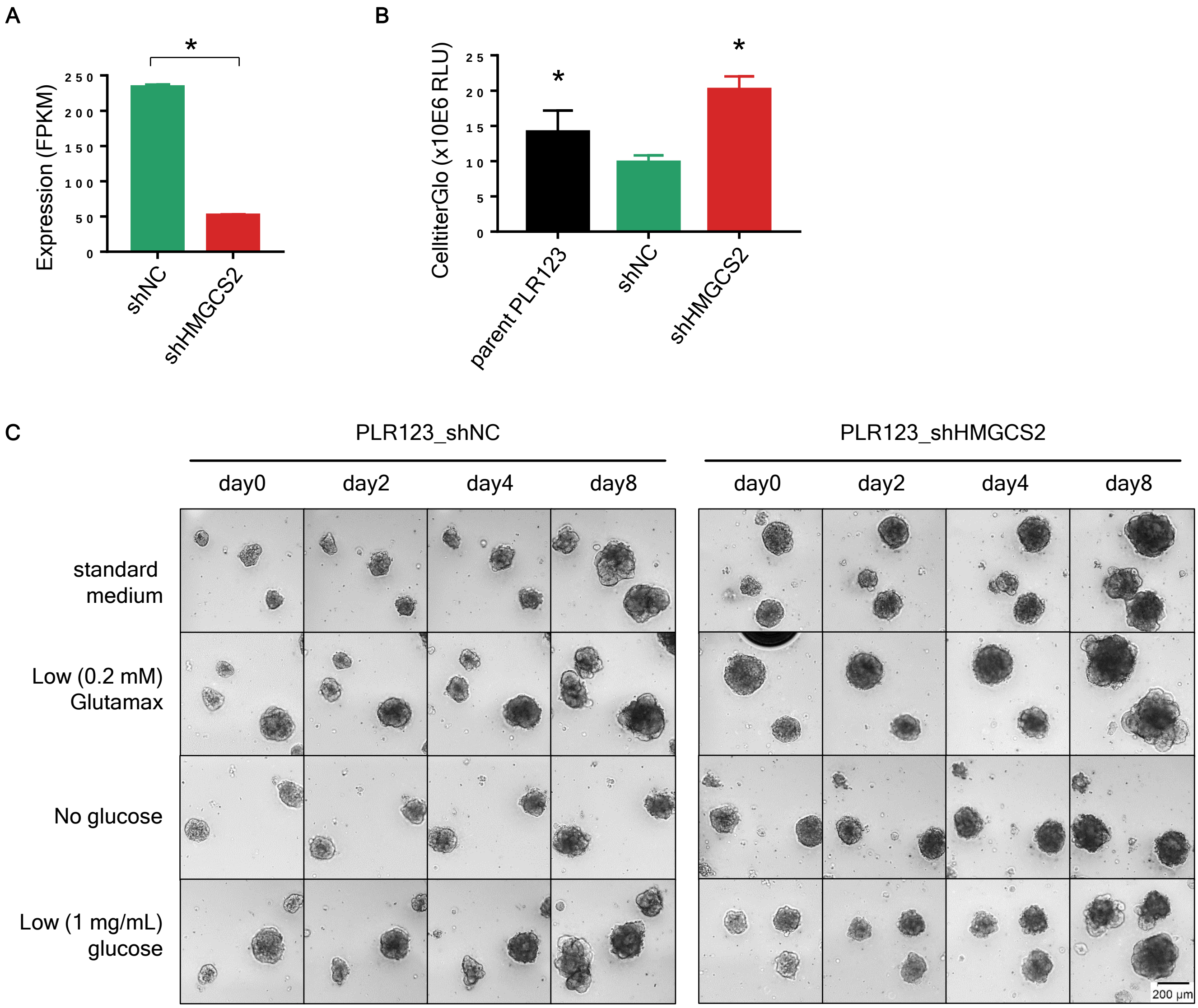

Figure S8. Effect of *HMGCS2* knockdown on regrowth. (A) HMGCS2 expression in *HMGCS2* knockdown cells (shHMGCS2) and control cells (shNC) in 2D cultures. Expression is indicated as FPKM obtained from RNA-seq data. **(B)** Cell viability in PLR123, shNC, and shHMGCS2 organoids in the regrowth assay (day10). *$P < 0.05$ vs. control group. Data are shown as mean ± standard deviation.**(C)** Bright-field images of entire PLR123, shNC, and shHMGCS2 organoids after SN-38 treatment in standard, low (0.2 mM) GlutaMAX, no glucose, or low (1 mg/mL) glucose condition. Scale bars, 200 µm.

Fig. S9

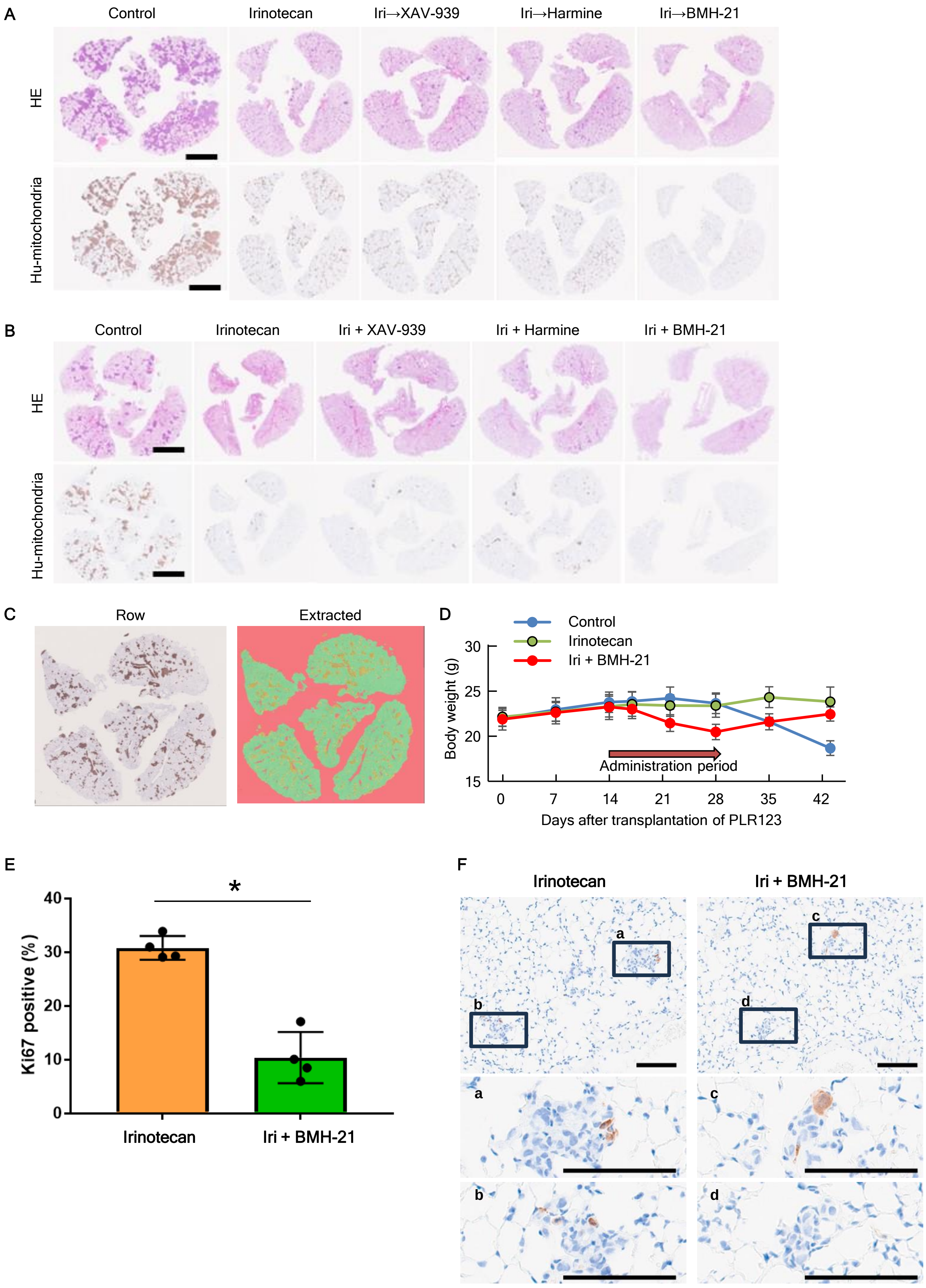
A
Control
Irinotecan
Iri→XAV-939
Iri→Harmine
Iri→BMH-21
HE
Hu-mitochondria
B
Control
Irinotecan
Iri + XAV-939
Iri + Harmine
Iri + BMH-21
HE
Hu-mitochondria
C
Row
Extracted
D
Control
Irinotecan
Iri + BMH-21
Body weight (g)
30
25
20
15
Administration period
0
7
14
21
28
35
42
Days after transplantation of PLR123
E
*
Ki67 positive (%)
40
30
20
10
0
Irinotecan
Iri + BMH-21
F
Irinotecan
Iri + BMH-21
a
b
c
d

Figure S9. Effects of chemical inhibitors in intravenous transplantation models with PLR123.

(A, B) Histopathological images of lungs in intravenous transplantation models established using PLR123 cells after sequential (A) and cycle (B) treatments. Hematoxylin–eosin and immunohistochemistry staining for human mitochondria. Scale bar, 5 mm. (C) Example of HALO AI image analysis in tumor nodules. Yellow, tumor nodule; green, lung tissue; red, background. (D) Body weight of mice in intravenous transplantation models administered cycle treatments. In the irinotecan and BMH-21 cycle treatment group, body weight decreased during the drug-administration period and increased during the drug-free period. The control group showed a decrease in body weight from day 35 following PLR123 transplantation due to tumor growth (E) Ki-67 positive rate in irinotecan and Iri+BMH-21 group in the experiment shown in Fig8L. *$P < 0.05$ (F) Histopathological images of Cleaved Caspase-3 for lungs in the experiment shown in Fig8L. Scale bars, 100 µm.

Fig. S10

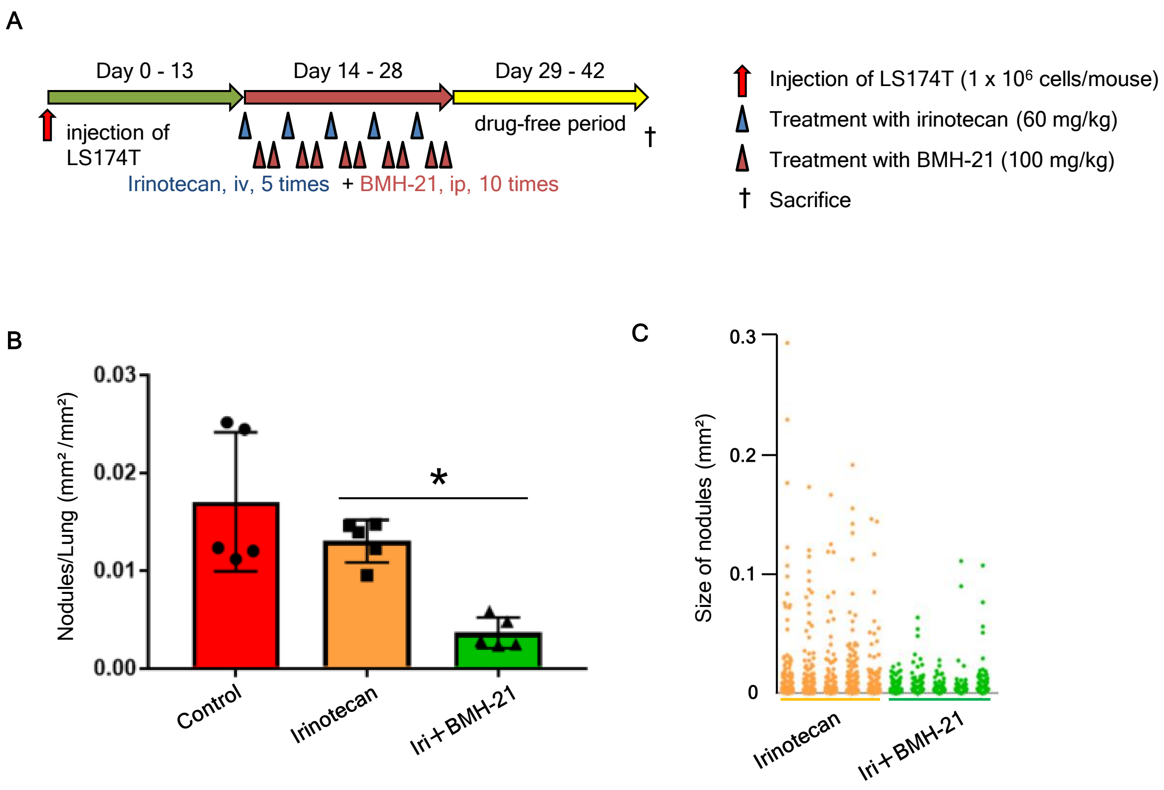

Figure S10. Effects of chemical inhibitor BMH-21 in intravenous transplantation models with LS174T.

(A) Study design for cycle treatment with irinotecan and BMH-21 using the intravenous transplantation model established using LS174T cells. Irinotecan monotherapy (60 mg/kg, iv) and BMH-21 monotherapy (100 mg/kg, ip, two times) were set as one cycle, and five cycle treatments were conducted. Lungs were sampled on day 42 (drug-free period) after the injection of LS174T cells (n = 5/group). Mice in the untreated control group were euthanized on day 27 because of deterioration of their general condition due to tumor masses in their systemic organs. (B) Image analysis of the total tumor nodule area. Data are shown as nodule area to lung tissue area (mm2/mm2) (mean ± standard deviation; each dot represents an individual value). (C) Image analysis of the sizes of all individual tumor nodules in a single sectioned tissue (mm2) (each dot represents an individual tumor nodule). *P < 0.05 vs. the irinotecan-only-treated group.
